# A chemical kinetic basis for measuring translation initiation and elongation rates from ribosome profiling data

**DOI:** 10.1371/journal.pcbi.1007070

**Published:** 2019-05-23

**Authors:** Ajeet K. Sharma, Pietro Sormanni, Nabeel Ahmed, Prajwal Ciryam, Ulrike A. Friedrich, Günter Kramer, Edward P. O’Brien

**Affiliations:** 1 Department of Chemistry, Pennsylvania State University, University Park, Pennsylvania, United States of America; 2 Centre for Misfolding Diseases, Department of Chemistry, University of Cambridge, Cambridge, United Kingdom; 3 Bioinformatics and Genomics Graduate Program, The Huck Institutes of the Life Sciences, Pennsylvania State University, University Park, Pennsylvania, United States of America; 4 Center for Molecular Biology of the Heidelberg University (ZMBH), DKFZ-ZMBH Alliance, Heidelberg, Germany; 5 German Cancer Research Center (DKFZ), Heidelberg, Germany; 6 Institute for CyberScience, Pennsylvania State University, University Park, Pennsylvania, United States of America; Tel Aviv University, ISRAEL

## Abstract

Analysis methods based on simulations and optimization have been previously developed to estimate relative translation rates from next-generation sequencing data. Translation involves molecules and chemical reactions, hence bioinformatics methods consistent with the laws of chemistry and physics are more likely to produce accurate results. Here, we derive simple equations based on chemical kinetic principles to measure the translation-initiation rate, transcriptome-wide elongation rate, and individual codon translation rates from ribosome profiling experiments. Our methods reproduce the known rates from ribosome profiles generated from detailed simulations of translation. By applying our methods to data from *S*. *cerevisiae* and mouse embryonic stem cells, we find that the extracted rates reproduce expected correlations with various molecular properties, and we also find that mouse embryonic stem cells have a global translation speed of 5.2 AA/s, in agreement with previous reports that used other approaches. Our analysis further reveals that a codon can exhibit up to 26-fold variability in its translation rate depending upon its context within a transcript. This broad distribution means that the average translation rate of a codon is not representative of the rate at which most instances of that codon are translated, and it suggests that translational regulation might be used by cells to a greater degree than previously thought.

## Author summary

The process of translating the genetic information encoded in an mRNA molecule to a protein is crucial to cellular life and plays a role in regulating gene expression. The translation initiation rate of a transcript is a direct measure of the rate of protein synthesis and is the key kinetic parameter defining translational control of the gene’s expression. Translation rates of individual codons play a considerable role in coordinating co-translational processes like protein folding and protein secretion and thus contribute to the proper functioning of the encoded protein. Direct measurement of these rates *in vivo* is nontrivial and recent next generation sequencing methods like ribosome profiling offer an opportunity to quantify these rates for the entire translatome. In this study, we develop chemical kinetic models to measure absolute rates and quantify the influence of different molecular factors in shaping the variability of these rates at codon resolution. These new analysis methods are significant because they allow scientists to measure absolute rates of translation from next-generation sequencing data, provide analysis tools rooted in the physical sciences rather than heuristic or *ad hoc* approaches, and allow for the quantitative, rather than qualitative study of translation kinetics.

## Introduction

Translation-associated rates influence *in vivo* protein abundance, structure and function. It is therefore crucial to be able to accurately measure these rates. The ribosome synthesizes a protein in three steps namely initiation, elongation, and termination [1–3]. Translation is initiated at the start codon of the mRNA transcript by the ribosomal subunits that form a stable translation-initiation complex [4,5]. During the elongation step, the ribosome moves along the mRNA transcript decoding individual codons and adding residues to the growing nascent chain. Translation is terminated when the stop codon is in the ribosome’s A-site resulting in release of the synthesized protein. The initiation and elongation phases of translation contribute to protein levels inside a cell; indeed, alteration of their rates can cause protein abundance to vary by up to five orders of magnitude [6–8], and alter protein structure and function [9]. Termination does not influence the cellular concentration of proteins as it is not a rate limiting step [10]. Therefore, knowledge of translation initiation and codon translation rates are important to understand the regulation of gene expression.

Significant efforts have been made to extract these rates from data generated from ribosome profiling experiments [11–14], a technique that measures the relative ribosome density across transcripts [15]. In a number of methods, the actual rates are not measured but instead a ratio of rates, or other relevant quantities have been reported [16–20]. Estimates of translation-initiation and codon translation rates have helped identify the molecular determinants of these rates. For example, estimated initiation rates correlate with the stability of mRNA structure near the start codon and in the 5' untranslated region [10,14,16,21] indicating mRNA structure can influence initiation. Similarly, codon translation rates have been found to positively correlate with their cognate tRNA abundance [16,22], and anti-correlate with the presence of downstream mRNA secondary structure [23,24] and positively charged nascent-chain residues inside the ribosome exit tunnel [20,25]. Some of these findings are controversial as different analysis methods and data have led to contradictory results concerning the role of tRNA concentration [16,19,26,27], positively charged residues [17,20] and coding sequence (CDS) length [10,12,14,16]. Moreover, the accuracy of these methods is unknown because orthogonal, high-throughput experimental measurements of translation rates do not exist.

In the absence of data that could differentiate the accuracy of different methods, we argue that the methods most likely to be accurate will be those that are constrained by and account for the chemistry and physics of the translating system. Here, we present three such methods, derived from chemical kinetic principles that permit the extraction of translation-initiation rates, transcriptome-wide average elongation rate and individual codon translation rates from Next-Generation Sequencing (NGS) data. These methods are verified against artificial ribosome profiling data generated from detailed simulations of the translation process where the translation rates are known *a priori*. We then apply these methods to *in vivo* ribosome profiling data and extract the transcriptome-wide translation-initiation and codon translation rates in *S*. *cerevisiae* and transcriptome-wide average elongation rate in mouse stem cells. We show that the translation rate parameters correlate with factors known to modulate these rates, and assign absolute numbers to these rates.

## Results

### Theory

#### Measuring translation-initiation rates

To derive an analytical expression relating the translation-initiation rate to the experimental observable of ribosome density along a transcript we assume steady state conditions, meaning that the ribosome flux at each codon position is equal to the rate at which fully synthesized protein molecules are released. Thus, for any transcript *i*, we have:

$$F(1,i)=F(2,i)=\cdots =F(j,i)=\cdots =F(N_{c}(i),i),$$ (1)

where *F*(1, *i*) is equal to the flux of ribosomes initiating translation at the start codon, *F*(*j*, *i*) is the flux of ribosomes moving from codon position *j* to (*j* + 1) on copies of transcript *i*, *N*_*c*_(*i*) is the number of codons in the transcript, and *F*(*N*_*c*_(*i*), *i*) is the flux of ribosomes terminating translation from the stop codon. In our modeling, the position of a ribosome on a transcript is defined by its A-site location.

Let l be the number of codon positions that a ribosome covers on a transcript. A ribosome typically covers 10 codon positions and hence *ℓ* = 10 with the A-site of the ribosome located at the sixth codon position in this segment [15]. A ribosome initiating translation will, therefore, cover codon positions 1 through 6 in the coding sequence, with the other portion covering part of the transcript’s 5′ UTR. It follows then that a ribosome translating any of the first l+1 codons will prevent a new ribosome from initiating translation by physically blocking the first six codon positions of the transcript. Thus, the ribosome flux at the start codon is

$$F(1,i)=\alpha (i)[1-\sum_{k=2}^{l+1}\rho (k,i)],$$ (2)

where *α*(*i*) is the initiation rate in the absence of any ribosome at the first l+1 codon positions, and *ρ*(*k*, *i*) is the average ribosome occupancy at the *k*^*th*^ codon position of the transcript [28]. Note well, Eq (2) assumes that the *ρ*(*k*, *i*)s for the first ten codon positions are independent of each other, and is therefore a first-order approximation of an exact solution and thus Eq (2) ignores higher order terms of *α*(*i*) [28]. This assumption may introduce inaccuracies in the calculation of *F*(1, *i*) for large *α*(*i*)s. Polysome profiling experiments [29] and computer simulations of protein synthesis [21], however, suggest that most *S*. *cerevisiae* transcripts are translated in the regime of low average ribosome density. As a consequence, ignoring higher order terms of *α*(*i*) in Eq (2) provides a reasonable estimate of *F*(1, *i*). Under a mean-field assumption [30], the ribosome flux at the *j*^*th*^ codon position for copies of transcript *i* can be written as

F(j,i)=ω(j,i)ρ(j,i)f(j,j+l,i). (3)

In Eq (3), *ω*(*j*, *i*) is the intrinsic translation rate of the *j*^*th*^ codon position in the transcript (*i*.*e*., the rate that would be observed in the absence of any other ribosomes), and f(j,j+l,i) is the conditional probability that given that a ribosome is at the *j*^*th*^ codon position there is no ribosome at the $({j+l)}^{th}$ codon position.

We used Eqs (1), (2) and (3) to derive (see S1 Text) the following expression for translation-initiation rate

$$\alpha (i)=\frac{\langle \rho (i)\rangle {(N}_{c}(i)-1)}{\langle T(i)\rangle [1-\sum_{k=2}^{l+1}\rho (k,i)]},$$ (4)

where $\langle \rho (i)\rangle =\frac{\sum_{j=2}^{N_{c}(i)}\rho (j,i)}{N_{c}(i)-1}$ is the average ribosome density per codon on the *i*^*th*^ transcript, and 〈*T*(*i*)〉 is the average time a ribosome takes to synthesize a full-length protein from the transcript. Eq (4) can measure the translation-initiation rate provided 〈*T*(*i*)〉, 〈*ρ*(*i*)〉 and the *ρ*(*j*, *i*)s are known. We calculated 〈*ρ*(*i*)〉 and *ρ*(*j*, *i*) from ribosome profiling, RNA-Seq and polysome profiling data, and 〈*T*(*i*)〉 using a scaling relationship [31] between protein synthesis time and CDS length. Full details for determining these parameters are provided in S1 Text.

#### Measuring the average translation elongation rate across a transcriptome

In ribosome run-off experiments performed on eukaryotic cells translation-initiation is inhibited at time point *t* = 0 using the drug harringtonine [26]. This causes ribosomes to accumulate at start codons and thereby block new translation-initiation events. At time *t* = Δ*t*, the cells are treated with cycloheximide, which stops translation elongation by the ribosomes. The experiment is repeated at different Δ*t* values. A meta-gene analysis of ribosome profiles [32] is then obtained from each run-off experiment and used to quantify the depletion of ribosome density across the transcripts as a function of time, allowing the transcriptome-wide average elongation rate to be measured [26].

To model this non-steady state data, we assume that ribosome movement along transcripts is a form of mass flow along one dimension–a reasonable assumption given that ribosomes can move only in one direction along a transcript. In this case, a natural way to describe this phenomenon is the continuity equation

$$\frac{d\rho }{dt}+\nabla ∙J=0,$$ (5)

which equates the decrease in density of a substance (*ρ*, the ribosome occupancy in our case) over time, *t*, with the outward flux ***J*** of that substance from a particular region of space. The equality with zero in Eq (5) is a manifestation of conservation of mass. In the ribosome run-off experiment this corresponds to a depletion of ribosome density along a segment of a transcript as ribosomes move out of that region with rate *J*, and are not replenished by new ribosomes since initiation was halted at time *t* = 0.

According to the definition of divergence, **∇** · ***J*** is the rate at which *ρ* exits from a given closed space [33]. For a system composed of *L* codons along a transcript that are being translated by ribosomes, $\nabla ∙J=\frac{J}{L}$ (see S1 Text, Eq. (S14)). Substituting this value of **∇** · ***J*** into Eq (5) and then integrating over *t* from 0 to Δ*t* yields

$$\frac{\rho (t=\Delta t,L)}{\rho (t=0,L)}=1-\frac{\Delta t}{\tau (L)},$$ (6)

where *ρ*(*t* = Δ*t*, *L*) is the average ribosome density of the transcriptome within the first *L* codon positions of a sample at run-off time Δ*t*. $\tau (L)=\frac{L\rho (t=0,L)}{J}$ is the average time at which $\frac{\rho (t=\Delta t,L)}{\rho (t=0,L)}$ equals zero. $\frac{\rho (t=\Delta t,L)}{\rho (t=0,L)}$ in Eq (6) is calculated using the ribosome run-off data. Explicit details of this calculation are provided in the S1 Text.

We inserted the relative average ribosome density *ρ*(*t* = Δ*t*, *L*)/*ρ*(*t* = 0, *L*) obtained from Eq (S16) into Eq (6) and plotted this against Δ*t*. By fitting this data to a straight line we determined *τ*(*L*), the time at which *ρ*(*t* = Δ*t*, *L*)/*ρ*(*t* = 0, *L*) equals zero. *τ*(*L*) is, therefore, the time at which the last translating ribosomes have crossed the *L*^*th*^ codon position, on average. We calculated *τ*(*L*) in this way for different *L* values up to the minimum *L* value at which ribosome density depletion no longer occurs even at the longest run off time in the experiment. The average transcriptome-wide elongation rate 〈*ω*〉 is therefore equal to

$$\langle \omega \rangle =\frac{dL}{d\tau (L)}.$$ (7)

#### Measuring individual codon translation rates

To derive a mathematical expression for extracting codon translation rates from ribosome profiling data we assumed steady state conditions in which the flux of ribosomes at each codon position is equal to the rate of protein synthesis

$$\frac{N_{2,i}^{ribo}}{\tau (2,i)}=\frac{N_{3,i}^{ribo}}{\tau (3,i)}=\cdots \frac{N_{j,i}^{ribo}}{\tau (j,i)}=\cdots =\frac{N_{N_{c}(i),i}^{ribo}}{\tau (N_{c}(i),i)}.$$ (8)

In Eq (8), $N_{j,i}^{ribo}$ and *τ*(*j*, *i*) are, respectively, the steady-state number of ribosomes and the average translation time of the *j*^*th*^ codon position within copies of transcript *i* in a given experimental sample. The mean total time of synthesis 〈*T*(*i*)〉 of transcript *i* is, by definition, equal to

$$\langle T(i)\rangle =\tau (2,i)+\tau (3,i)+\cdots +\tau (N_{c}(i),i),$$ (9)

Solving Eqs (8) and (9) for *τ*(*j*, *i*) (see derivation in S1 Text) yields

$$\tau (j,i)=\frac{N_{j,i}^{ribo}}{\sum_{l=2}^{N_{c}(i)}N_{l,i}^{ribo}}\langle T(i)\rangle .$$ (10)

As is the convention in the field [34], we assume that ribosome profiling reads at the *j*^*th*^ codon position of transcript *i*, *c*(*j*, *i*), are directly proportional to $N_{ij}^{ribo}$. This relationship can be expressed as

$$N_{j,i}^{ribo}=a_{j,i}c(j,i),$$ (11)

where *a*_*j*,*i*_ is a constant of proportionality. *a*_*j*,*i*_ values have not been experimentally measured, but they are commonly assumed to be constant for all codon positions in a single experiment [34]. That is, *a*_*j*,*i*_ = *a*_*i*_ for all *i* and *j*. Using Eq (11) with *a*_*j*,*i*_ = *a*_*i*_ in Eq (10) yields

$$\tau (j,i)=\frac{c(j,i)}{\sum_{l=2}^{N_{c}(i)}c(l,i)}\langle T(i)\rangle .$$ (12)

Eq (12) indicates that we can determine the individual codon translation rates from ribosome profiling reads provided we know the average total synthesis time of the transcript. Eq (12) can be connected to the expression for normalized ribosome density, derived in the SI of Ref. [16], where $\frac{\tau (j,i)}{\tau (i)}N_{c}(i)$ is the normalized ribosome density and is expressed as a function of *c*(*j*, *i*)s. Eq (12) is also related to a metric used in the simulations of Ref. [14] to estimate the codon translation rates. It is important to note that *τ*(*j*, *i*) is the actual codon translation time, which includes the time delay caused by ribosome-ribosome interactions and is distinct from the intrinsic translation rate of a codon *ω*(*j*, *i*). *τ*(*j*, *i*) is equal to the inverse of ω(j,i)f(j,j+l,i) [35].

### Application

#### *In silico* validation of our methods

**Fig 1:**
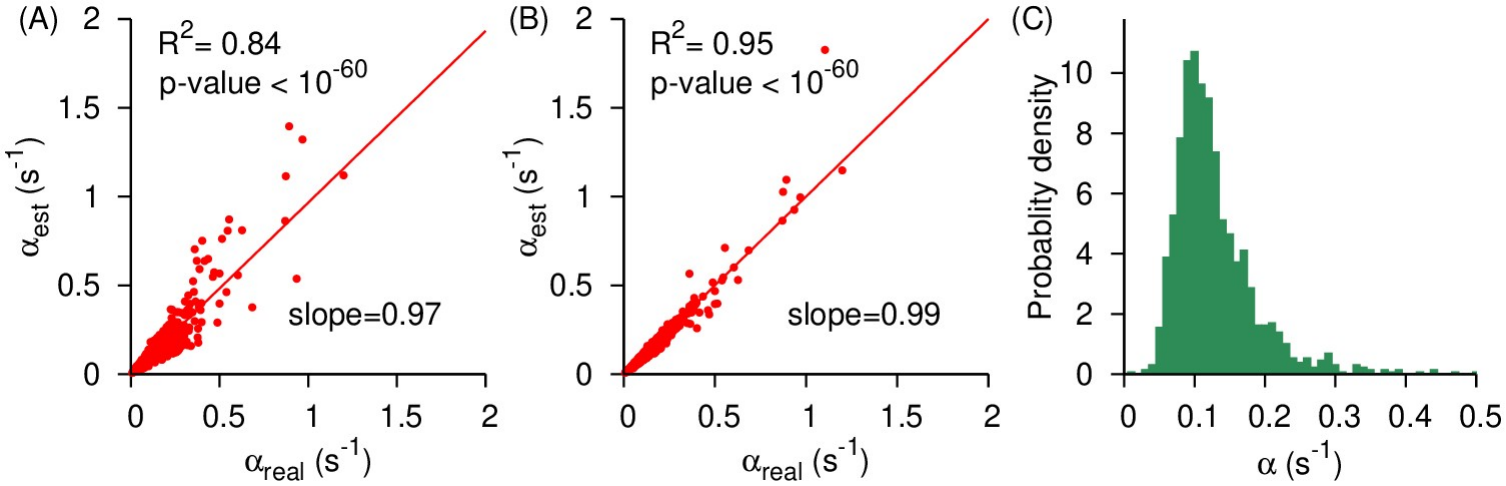
Eq (4) accurately determines the translation-initiation rate from simulated *S*. *cerevisiae* ribosome profiles and its application to experimental data. (**A**) Translation-initiation rates determined by applying Eq (4) to simulated ribosome profiling data are plotted against the actual initiation rates used in the simulations. These initiation rates were calculated using Eq (S10) for the average protein synthesis times. (**B**) Same as (**A**) but the average protein synthesis times were measured from our simulations of the translation process. The solid lines in (**A**) and (**B**) are the lines of the best fit. (**C**) The distribution of *in vivo* translation-initiation rates measured by applying Eq (4) to experimental data involving 1,287 *S*. *cerevisiae* high coverage transcripts.

**Fig 2:**
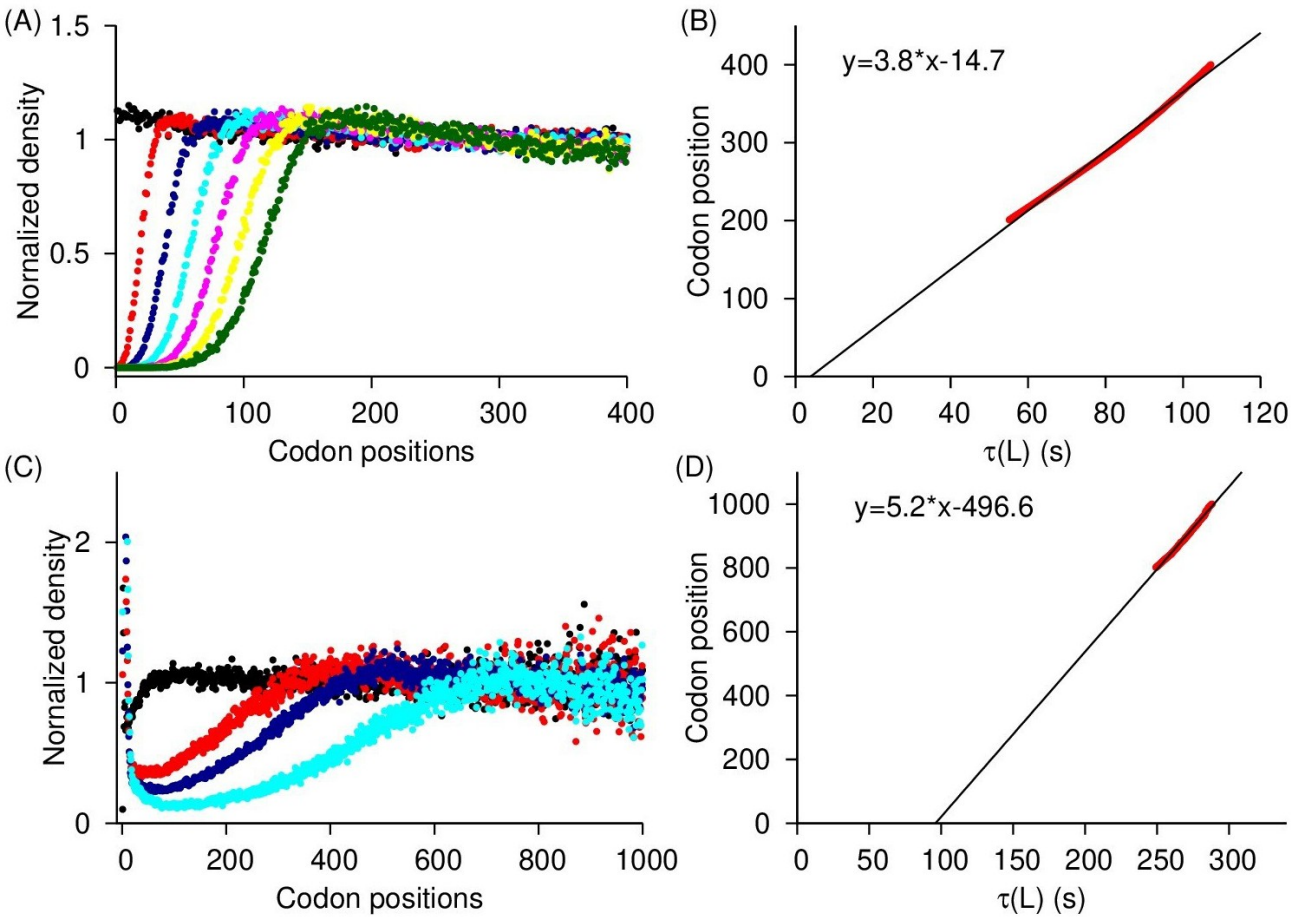
Measuring average elongation rate by applying Eq (7) to *in silico* and *in vivo* ribosome run-off data. (**A**) Normalized average ribosome read density ($\overset{-}{R_{T}}(j,\Delta t)$), Eq. (S15), calculated from simulated ribosome run-off experiment is plotted as a function of codon position for run-off times of 0, 5, 10, 15, 20, 25 and 30 *s*^−1^ with black, red, blue, cyan, pink, yellow and green data points, respectively. (**B**) The average time taken to fully deplete the normalized average ribosome read density within a window of the most 5′ codons positions in *S*. *cerevisiae* transcripts are plotted against the most 3′ codon position of the window. (**C**) Normalized average ribosome read density, calculated from *in vivo* run-off experimental data reported in Ref. [26], are plotted as a function of codon position for the run-off times of 0, 90, 120 and 150 seconds with black, red, blue and cyan data points, respectively. (**D**) The average time taken to fully deplete the normalized average ribosome read density within a window of the most 5′ codons positions in mouse stem cells transcripts are plotted against the most 3′ codon position of the window. The negative intercept reflects the time taken by harringtonine to engage with ribosomes.

**Fig 3:**
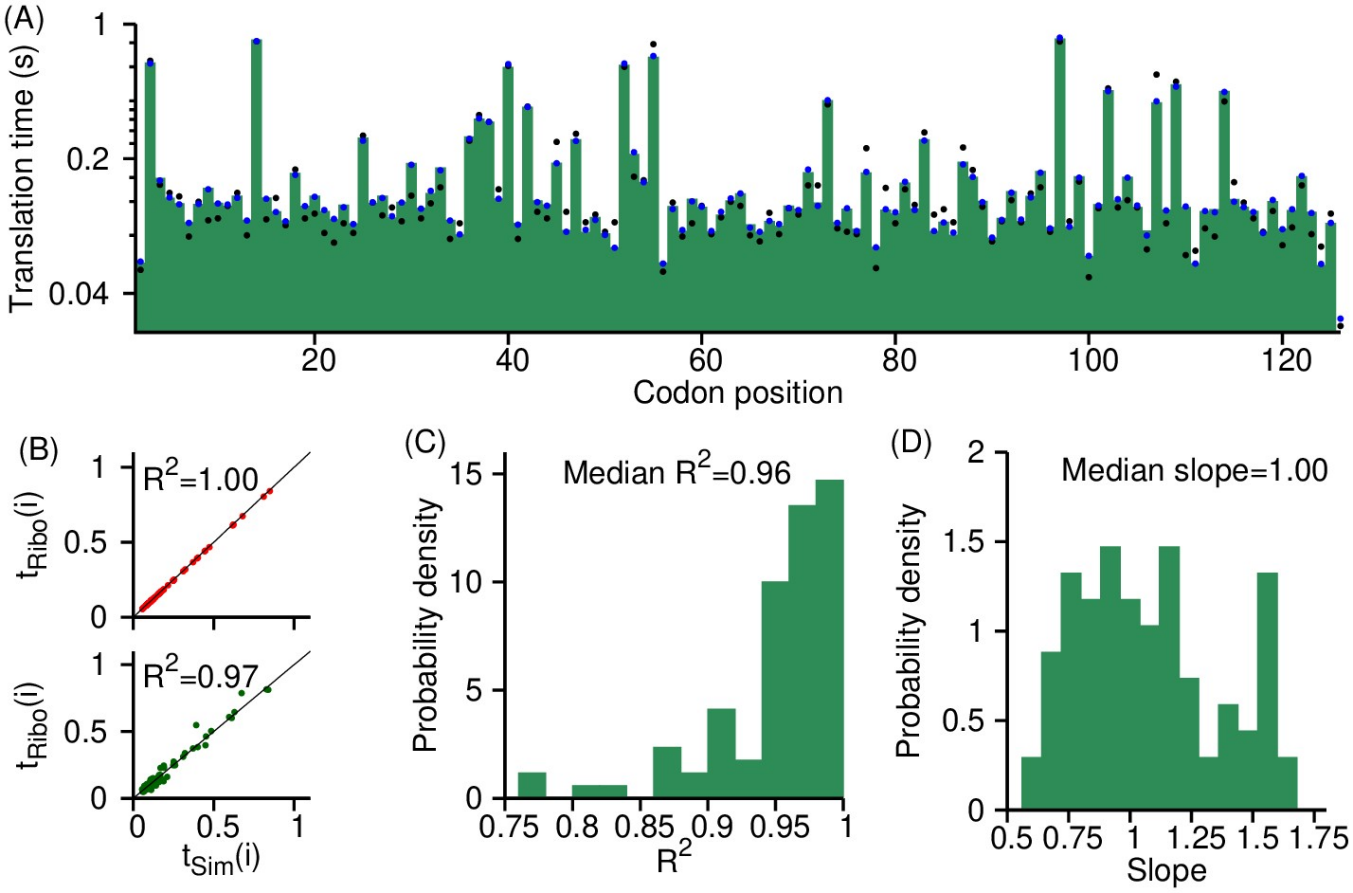
Eq (12) accurately determines codon translation times from simulated ribosome profiles. (**A**) Average translation time of a codon in YER009W *S*. *cerevisiae* transcript is plotted as a function of its position within the transcript. The true codon translation times in the simulations are plotted as green boxes, blue and black data points are the translation times measured using Eq (12). Blue data points were calculated using the average protein synthesis time measured from the simulations and relative ribosome density calculated using a large number of *in silico* ribosome profiling reads. Black data points were calculated using the average protein synthesis time estimated from the scaling relationship (Eq. (S10)) and the relative ribosome density calculated from the *in silico* reads which were equal to the reads aligned to the same transcript in the experiment [16]. (**B**) Measured codon translation times, plotted with black and blue data points in (**A**), are plotted against true codon translation times in the simulations in the top and bottom panel, respectively. (**C**) Probability distribution of the *R*^2^ correlation between the true and calculated codon translation times for the 85 *S*. *cerevisiae* transcripts. (**D**) Probability distribution of the slope of the best-fit lines between the estimated and true codon translation times for the 85 *S*. *cerevisiae* transcripts. The high *R*^2^ in (**C**) and median slope of 1.00 in (**D**) indicate that Eq (12) can, in principle, accurately measure absolute rates.

As a first step to test the accuracy of the measured translation rates from Eqs (4), (7) and (12), we applied them to artificial *S*. *cerevisiae* ribosome profiles generated by kinetic Monte Carlo [36] and Gillespie simulations [37] in which all of the underlying rates are known (see Methods). If our analysis methods are accurate then a necessary condition is that they reproduce these rates from the simulated profiles. We applied Eq (4) to the simulated, steady-state ribosome profiling data and find that it quantitatively reproduces the initiation rates used in the simulations (Fig 1A, slope = 0.97, *R*^2^ = 0.84 and p-value < 10^−60^). We applied Eq (7) to the non-steady state ribosome run off profiles of *S*. *cerevisiae* (Fig 2A and 2B) and find that it accurately measures the transcriptome-wide average translation rate. Specifically, our method measures the transcriptome-wide average elongation rate at 3.8 AA/s against the real average elongation rate of 4.2 AA/s. Note well, that as predicted by Eq (6), we also observe a linear decrease in $\frac{\rho (t=\Delta t,L)}{\rho (t=0,L)}$ as a function of time (S1A Fig). Finally, we applied Eq (12) to the steady-state ribosome profiles and find that the individual codon translation times are accurately measured by our method (Fig 3, median *R*^2^ = 0.96 and median slope = 1.00). Thus, the analysis methods we have created can in principle accurately capture the translation rate parameters.

There are several points worth noting concerning these tests. First, the rates used in the simulation model are realistic, having been taken from literature values [10,38]. Second, the depth of coverage in the simulated ribosome profiles is in the same range as experiments, *e*.*g*., having 26 million reads arising from 1,388 different coding sequences [16]. Third, Eqs (4) and (12) require knowledge of the average synthesis time of a protein, which is experimentally difficult to measure. Therefore, in the above analyses we used the approximation that the average synthesis time of a protein is proportional to the number of codons in its transcript, multiplied by the transcriptome-wide average codon translation time (Eq. (S10)) [26,31]. However, when we use the actual synthesis time of each transcript we achieve even better agreement between the estimated and true translation initiation rates (Fig 1B, slope = 0.99, *R*^2^ = 0.95 and p-value < 10^−60^). Similarly, when we increase our read coverage from 7.1 million to 35.5 billion reads and use the exact synthesis time of a protein, *R*^2^ between the estimated and true codon translation rates goes to > 0.99. Thus, our model is reasonably accurate when approximate protein synthesis times are used (Eq. (S10)) and the coverage is similar to typical experiments, and highly accurate when the exact synthesis time is used and coverage is high.

Ribosome profiling data tend to exhibit a higher ribosome density near the start codon as compared to the rest of the transcript [16]. This feature might bias the *in vivo* measurement of initiation rate using our method, as Eq (4) is a function of the ribosome density of the first 10 codon positions. To assess the effect of this feature on the accuracy of our method we created *in silico* ribosome profiles by running synthesis simulations in which the translation rates of the first 100 codons were 50% slower for all *S*. *cerevisiae* 1,388 transcripts. This artificial decrease in codon translation rates introduced more ribosome density near the start codon. Using these *in silico* ribosome profiles, we find that Eq (4) still recapitulates the initiation rates with a similar level of accuracy as before (S2 Fig, *R*^2^ ≥ 0.85). Thus, higher ribosome density near the start codon does not substantially affect the accuracy of our method. Our simulations do not account for other possible biases associated with ribosome profiling experiments, including sequencing amplification biases [39]. Thus, the accuracy of our model as determined from these *in silico* ribosome profiles might represent an upper bound to the accuracy on actual *in vivo* profiles with similar sequencing depth.

#### Measuring the initiation rate from experimental data

We next applied Eq (4) to *in vivo* ribosome profiling and RNA-Seq data from *S*. *cerevisiae* reported in Ref. [16]. Calculation of the translation-initiation rate for a transcript requires knowledge of the average time a ribosome takes to synthesize the protein (〈*T*(*i*)〉) and the average occupancies of the first ten codon positions (*ρ*(*j*, *i*)). The average protein synthesis time was calculated using the scaling relationship (Eq. (S10)) where the transcriptome-wide average codon translation time was set to 200 ms as reported in the literature [40,41] (see S1 Text). *ρ*(*j*, *i*)s were calculated from Eq. (S9) using the ribosome profiling and RNA-Seq data from Ref. [16] and polysome profiling data from Ref. [42]. We then inserted these arguments into Eq (4) to calculate the translation-initiation rates for 1,287 *S*. *cerevisiae* transcripts that meet the filtering criteria (see Methods) and for which polysome profiling data is available. We find the translation-initiation rates vary from as low as 5.8 × 10^−2^ *s*^−1^ (5^th^ percentile) to as high as 0.24 *s*^−1^ (95^th^ percentile) with the most probable rate being 0.1 *s*^−1^ (Fig 1C and S1 File). These translation-initiation rates are significantly lower than the average elongation rate in *S*. *cerevisiae* which is consistent with previous studies indicating that initiation is the rate limiting step in translation [21]. The distribution of translation-initiation rates measured using Eq (4) is very similar to the distribution reported in Ref. [10], which was obtained using polysome profiling data.

To test the reproducibility of these results we calculated *in vivo* translation-initiation rates using the ribosome profiling and RNA-seq data measured by Nissley *et al*. [43] and polysome profiling data in Refs. [29,42]. These *in vivo* initiation rates are reported in the S1 File. We have found a statistically significant correlation between the initiation rates measured from the Weinberg *et al*. [16] and Nissley *et al*. [43] data sets (S3 Fig). The Pearson correlation between them was 0.53 (*P* = 10^−46^) and 0.31 (P = 4 × 10^−15^) when polysome profiling data of Mackay *et al*. [42] and Arava *et al*. [29] were used, respectively. We have also compared these *in vivo* initiation rates with the ones measured in the study of Dao Duc and Song [14] and found a statistically significant correlation between them with Pearson *r* varying from 0.51 to 0.80 (*P* < 10^−24^), depending upon the data sets used (S4 Fig).

#### Reproducing known correlations with initiation rates

**Fig 4:**
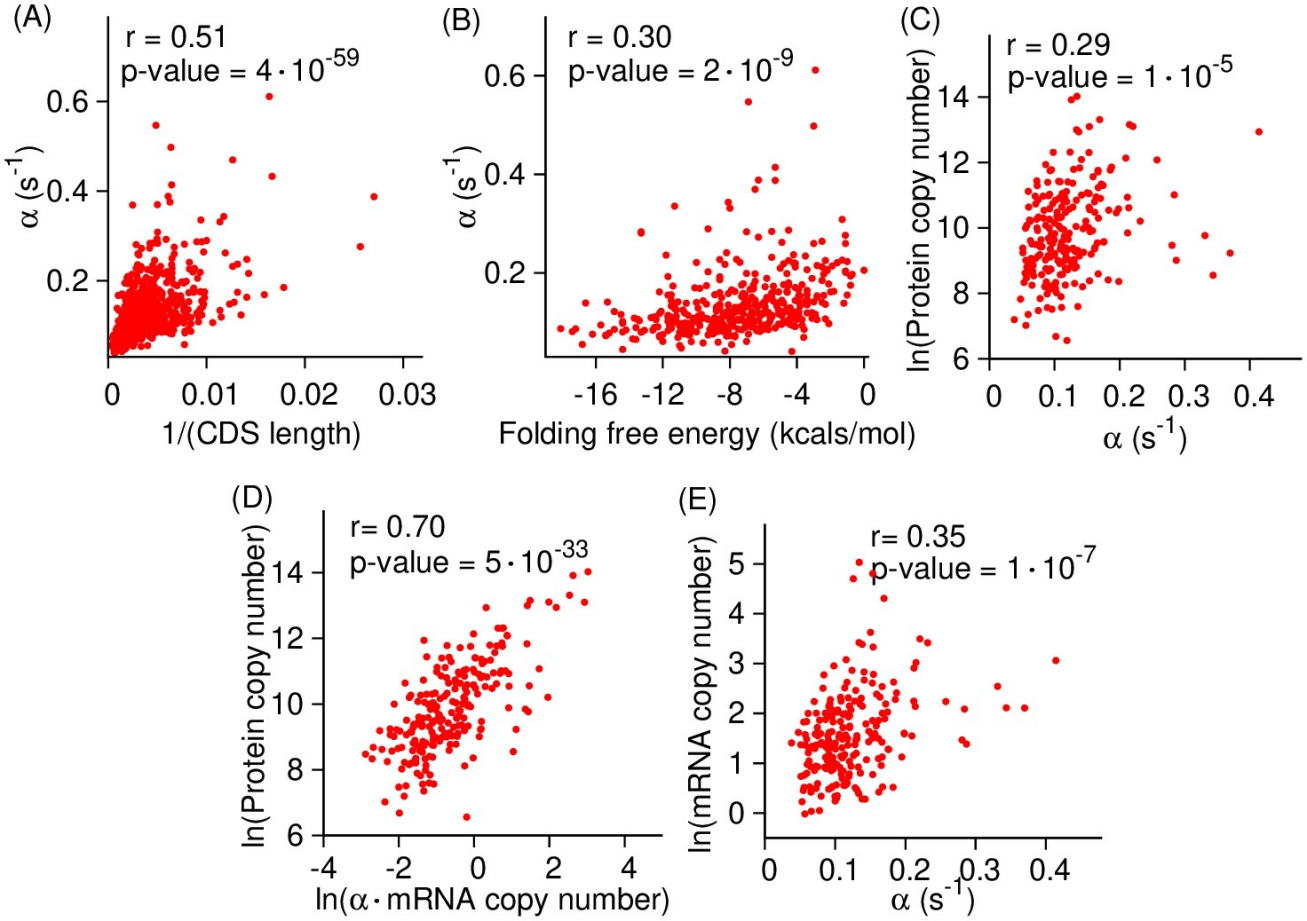
Translation-initiation rates measured using Eq (4) reproduce previously reported correlations with molecular properties. *In vivo* translation-initiation rates of *S*. *cerevisiae* transcripts are plotted against the inverse of their CDS length, folding energy of mRNA molecule near the 5′ cap and protein copy number in (**A**), (**B**) and (**C**), respectively. (**D**) The copy number of *S*. *cerevisiae* proteins are plotted as a function of the product of the initiation rate of the transcripts that encode them and that transcript’s copy number in a cell. (**E**) mRNA copy number is plotted against the translation-initiation rate.

Next, we tested whether our measurements reproduce previously reported correlations between initiation rates and CDS length, sequence context upstream and around the start codon, folding energy near the 5′ cap of mRNA molecule, and protein copy number. Initiation rates can depend on CDS length because the shorter a transcript, the more probable the termini will be in close proximity, allowing more efficient re-initiation of terminating ribosomes [44]. We used the initiation rates extracted from ribosome profiling and RNA-Seq data reported in Ref. [16], and polysome profiling data reported in Ref. [42] and find a moderate but statistically significant correlation (Pearson *r* = 0.51, p-value = 4 × 10^−59^) between the translation-initiation rate and the inverse of CDS length (Fig 4A), as has been observed previously [10,14,16].

Folded mRNA structure near the 5′ cap can disrupt the binding of initiation factor eIF4F and the scanning of 40S ribosomes in eukaryotes, causing a decrease in a gene’s translation-initiation rate [14,16,45,46]. We calculated the mRNA folding energy near the 5′ mRNA cap (see Methods) and find a statistically significant correlation between them and our translation-initiation rates (Fig 4B, Pearson *r* = 0.30 and p-value = 2 × 10^−9^).

Sequence-based features also determine the rate of translation-initiation in eukaryotes. For example, an upstream open reading frame can potentially interfere with translation initiation at the canonical start codon by initiating translation prematurely resulting in a different protein product [47,48]. We tested whether upstream open reading frames affect initiation rates by comparing the median initiation rate in transcripts with at least one upstream AUG site against transcripts that do not contain any upstream AUG. We find that the transcripts with at least one upstream AUG codon have a median initiation rate that is 15% slower (0.095 *s*^−1^ versus 0.112 *s*^−1^, Mann-Whitney U test, p-value = 0.006). Weinberg *et al*. [16] also demonstrated this effect with their measure of initiation efficiency. Additionally, the 12-nt Kozak sequence [49] is enriched in highly expressed genes and mutations in the Kozak sequence have been shown to drastically effect protein abundance levels [50]. We find that initiation rates are 16% faster with a median value of 0.116 *s*^−1^ in transcripts with Kozak-like sequences around the start codon as compared to those that do not contain these mRNA sequence motifs (Mann-Whitney U test, p-value = 0.005). Hence, the presence of Kozak or Kozak-like sequences around the start codon facilitate translation initiation. This is consistent with results of Pop *et al*. [13] who have shown a strong correlation between the presence of the Kozak sequence and translation efficiency.

Initiation is typically the rate limiting step of translation [21]. Therefore, a faster initiation rate should increase the rate of protein synthesis and thus the steady-state level of proteins in a cell. Indeed, we find a statistically significant correlation between the rate of translation-initiation and protein copy number in a cell (Fig 4C, Pearson *r* = 0.29 and p-value = 1 × 10^−5^). Next, we tested whether the missing variation in Fig 4C is explained by the variation in mRNA copy number. We did this by measuring the correlation between the protein copy number and the multiplicative product of a transcript’s copy number and its initiation rate. We find a stronger correlation between this quantity and protein copy number (Fig 4D, Pearson *r* = 0.70 and p-value = 5 × 10^−33^). These results suggest, as has been found previously [21,51], that the translation-initiation rate is a major determinant of protein abundance inside a cell.

We further measured the correlation between the mRNA copy number and initiation rates and found a moderate level of correlation between them (Fig 4E, Pearson *r* = 0.35 and p-value = 1 × 10^−7^). This suggests that higher copy number transcripts have a faster translation-initiation rate, consistent with previous studies [52]. We also find statistically significant correlations between the aforementioned quantities when we calculated the translation-initiation rate from other published data, using all possible combinations of ribosome profiling, RNA-Seq [16,43] and polysome profiling datasets [29,42] (S5–S7 Figs, S1 and S2 Tables).

In summary, we find that the initiation rates we measured correlate with factors that have been established to influence translation speed. This suggests our initiation rates are reasonable.

#### Measurement of the average codon translation rate in a cell

Next, we applied Eq (7) to measure the average codon translation rate inside mouse stem cells from ribosome run-off experiments [26]. We calculated the average normalized reads $\overset{-}{R_{T}}(\Delta t,j)$ from three different samples prepared by allowing the ribosomes to continue their elongation for 90, 120, and 150 seconds after the inhibition of initiation, and plotted $\overset{-}{R_{T}}(\Delta t,j)$ as a function of codon position (Fig 2C). By following the analysis procedure described in the Theory Section, we fitted $\frac{\rho (t=\Delta t,L)}{\rho (t=0,L)}$ against Δ*t* curves (S1B Fig) with a line and calculated the depletion time *τ*(*L*), where $\frac{\rho (t=\Delta t,L)}{\rho (t=0,L)}$ equals zero for *L* varying between codon positions 800 and 1000. Plotting *τ*(*L*) against *L* yields a straight line (Fig 2D). The gradient of this line is the average elongation rate, which we find equals 5.2 AA/s.

#### Measurement of individual codon translation rates

To extract individual codon translation times along a coding sequence we applied Eq (12) to 117 and 364 high-coverage transcripts from ribosome profiling data reported, respectively, in Refs. [43] and [53] (see Methods). The number of transcripts in both of these datasets are small as compared to the size of *S*. *cerevisiae* transcriptome. Therefore, to determine whether these subset of transcripts are representative of the whole transcriptome we compared the distributions of different physicochemical properties in these two sets to the total transcriptome. We find that the subset of transcripts from Nissley *et al*. [43] have 6.6% higher mean GC content but a very similar mode of length distribution and codon usage relative to the total transcriptome (S8A–S8C Fig). For Williams *et al*.’s ribosome profiling dataset [53] we again find that the mode of the length distribution and codon usage is similar to the *S*. *cerevisiae* transcriptome, with 5.3% higher mean GC content (S8D–S8F Fig). This indicates that the set of transcripts we analyze are largely representative of the properties of the transcriptome, but have a bit higher GC content.

**Fig 5:**
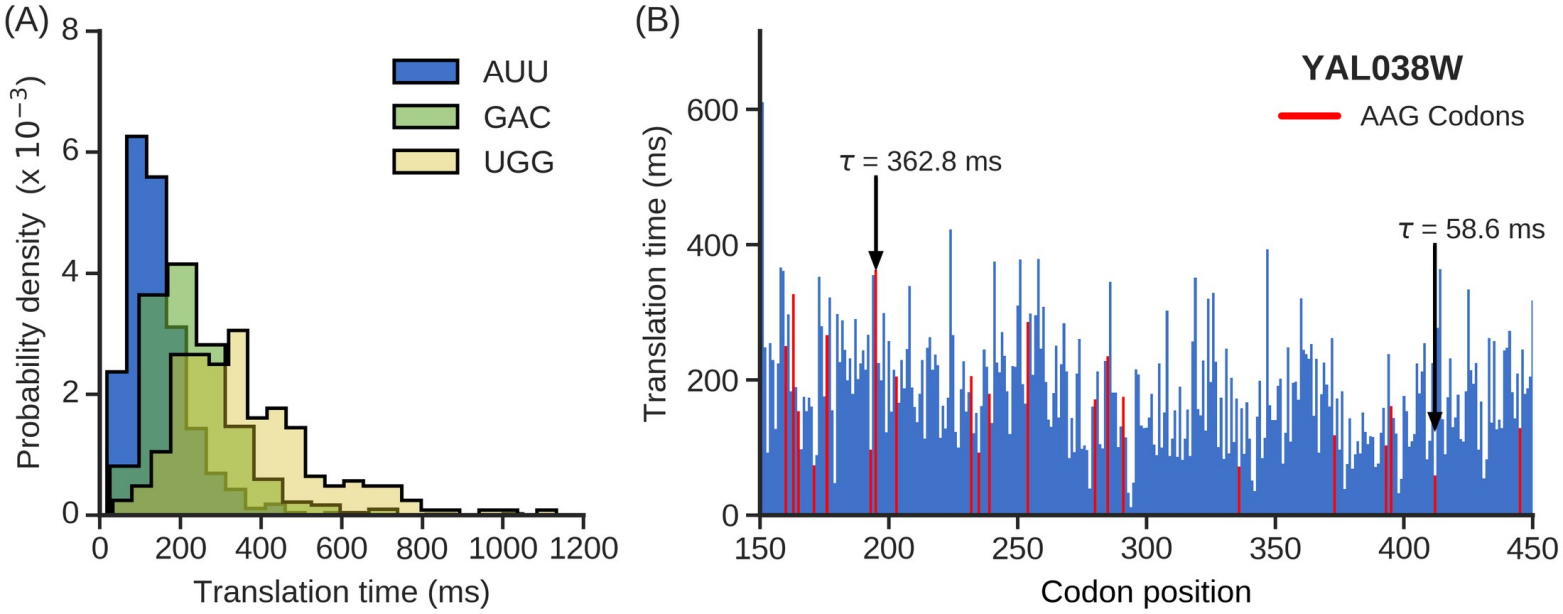
Wide variability in individual codon translation rates *in vivo*. **(A)** Probability density functions for translation times of AUU, GAC and UGG codons in Nissley dataset. Median translation times for AUU, GAC and UGG codon are 127, 208 and 331 ms, respectively. (**B**) The translation time profile *of S*. *cerevisiae* transcript YAL038W from Nissley dataset is shown between codon positions 150 and 450. AAG codon (colored red) is translated in 362.8 ms at the 196^*th*^ codon position and in 58.6 ms at 413^*th*^ codon position.

Upon extracting individual codon translation times from these ribosome profiling data, we first characterized the distribution of translation times for the 61 sense codons (S1 File). We find around three-fold difference between the median translation times of the fastest and slowest codons in the Nissley dataset [43]. The fastest and slowest codons are AUU and CCG codons that are translated in 127±2 and 344±37 ms (median ± standard error), respectively. The variability in translation times for a given codon type is even larger, as illustrated by wide distributions of their translation times in the Nissley dataset (Fig 5A, S9A Fig). Fig 5B shows an example where the AAG codon is translated with translation times ranging from 59 ms at codon position 413 to 363 ms at codon position 196 in YAL038W *S*. *cerevisiae* transcript. We find a 16-fold variability in codon translation times across the transcriptome even if we ignore the extremities of the distributions by only considering the translation times between the 5^th^ and 95^th^ percentiles of all codon types. Similar ranges are found in the Williams dataset where there is a 26-fold variability in translation times and 3.9 fold-difference in median translation times of the fastest (AUC) and slowest (CGA) codons, which are translated with median time of 128±2 and 496±61 ms, respectively (S1 File, S9B Fig). The translation time distributions are well correlated between the above two datasets (S10A and S10B Fig). The study of Dao Duc and Song [14] also infers the individual codon translation rates and a very high correlation is observed between the rates obtained using our method and the rates found in that study (S10C Fig).

#### Molecular factors flanking the A-site shape the variability of individual codon translation rates

**Fig 6:**
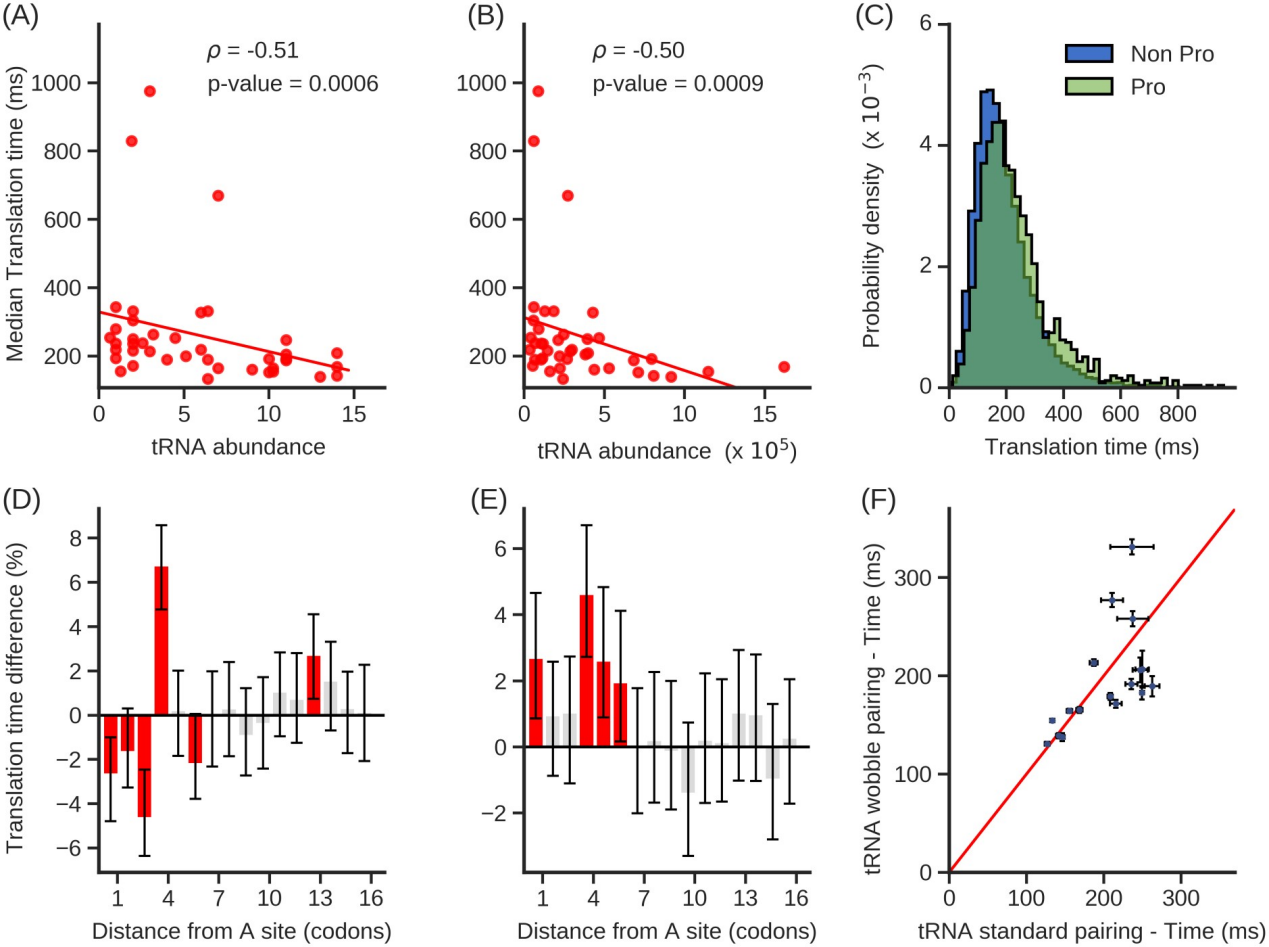
Molecular factors shaping the variability of individual codon translation rates. **(A-B)** Median translation times of codon types are negatively correlated with cognate tRNA abundance estimated by **(A)** gene copy number and **(B)** RNA-Seq gene expression. **(C)** Probability distribution of translation times of codons in the A-site either when a proline is present in the P-site (green) or when a proline is not present in the P-site (blue). **(D-E)** Percentage difference in median translation times when mRNA structure is present relative to when it is not present is plotted as a function of codon position after the A-site. Grey bars indicate results that are not statistically significant. Error bars are the 95% C.I. calculated using 10^4^ bootstrap cycles; significance is assessed using the Mann-Whitney U test corrected with the Benjamini Hochberg FDR method for multiple-hypothesis correction. mRNA structure information used in (**D**) and (**E**) are provided by *in vivo* DMS and *in vitro* PARS data, respectively. **(F)** Scatter plot of the median translation times of pairs of codon types that are decoded by the same tRNA molecule. The red line is the identity line. The list of tRNA molecule names and decoded codon types were taken from Ref. [54]. Error bars are standard error about the median translation time calculated with 10^4^ bootstrap cycles.

A number of molecular factors have been shown or suggested to influence the translation rate of a codon in the A-site, including tRNA concentration, mRNA structure, wobble-base pairing, and proline residues at or near the ribosome P-site [16,18–20,22–24,39]. Here, we test whether the presence or absence of these factors correlate with changes in translation speed that we measure. We first examined whether the cognate tRNA concentration correlates with our translation times. We find that the median codon translation times negatively correlates with the abundance of cognate tRNA (Fig 6A and 6B, *ρ* = −0.51 (p-value = 0.0006) and *ρ* = −0.50 (p-value = 0.0009), respectively), indicating that codons with lower cognate tRNA concentrations typically are translated more slowly.

The presence of a proline amino acid at the ribosome’s P-site can slowdown translation due to its stereochemistry [55]. We tested whether such an effect was present in our data set by calculating the percentage difference in median translation time at the A-site when proline is present at the P-site versus when it is not present at the P-site. We find a 19% increase in median translation time when proline is present (Fig 6C, Mann-Whitney U test, p-value = 2.2 × 10^−32^) indicating that proline does systematically slowdown translation *in vivo*.

It has been found that the presence of downstream mRNA secondary structure can slow down the translation at the A-site [23,24,56,57]. To test for this effect, we plotted the difference in the median translation time at the A-site when mRNA secondary structure is present versus when it is not present at a given number of codon positions downstream of the ribosome A-site. Structured versus unstructured nucleotides were identified using DMS data [58]. We find that when secondary structure is present 4 codons downstream of the A-site, placing that structure directly at the leading edge of the ribosome, there is on average a 6.7% increase in codon translation time at the A-site (Fig 6D, Mann-Whitney U test, p-value = 2.7 × 10^−14^). A slowdown is also found when we cross-reference our codon translation times with mRNA structure data from PARS [59], which measures the presence of mRNA structure *in vitro* (Fig 6E, Mann-Whitney U test, p-value = 5.6 × 10^−9^).

Wobble base pairing between the codon and anti-codon tRNA stem-loop has been found to slowdown translation speed as compared to Watson-Crick base pairing in bacteria [60] and metazoans [61]. For each pair of codon types that are decoded by the same tRNA molecule, by Watson-Crick base pairing in one instance and wobble base pairing in the other, we tested whether two codon types are translated with different rates. We find that there is no systematic difference in median translation times between codons that are decoded by either mechanism (Fig 6F, Wilcoxon signed-rank test, p-value = 0.46), indicating that, at least in *S*. *cerevisiae*, wobble base pairing does not slowdown *in vivo* translation elongation.

These results were reproduced using another dataset [53] that also shows that codon translation times anti-correlate with tRNA concentration (S11A and S11B Fig, *ρ* = −0.58 (p-value = 7.8 × 10^−5^) and *ρ* = −0.56 (p-value = 0.0002), respectively), exhibit larger translation times when a proline is present at the P-site (S11C Fig, Mann-Whitney U test, p-value = 3.0 × 10^−27^) or when mRNA structure is present downstream of the A-site (S11D and S11E Fig, Mann-Whitney U test, p-values = 3.6 × 10^−5^, 8.7 × 10^−4^, respectively) and similarly, we found no difference between the translation rate of codons that are translated with Watson-Crick and Wobble base pairing (S11F Fig, Wilcoxon signed-rank test, p-value = 0.88).

## Discussion

We have presented three methods for measuring initiation rates and elongation rates from ribosome profiling data. What distinguishes our approach from many others is that it uses simple equations derived from chemical kinetic principles, it does not require simulations or a large number of parameters, and it yields absolute rather than relative rates. We demonstrated that our approach provides accurate results when applied to test data sets (Figs 1, 2 and 3), and reproduced previously reported correlations between translation speed and various molecular factors (Figs 4 and 6, S5–S7 and S11 Figs), suggesting the rates obtained by these methods are reasonable.

A novel finding concerning elongation rates is that in *S*. *cerevisiae* the translation time of a codon depends dramatically on its context within a transcript. In *S*. *cerevisiae*, the range of individual codon translation time spans up to 26-fold, from 45 to 1,194 ms, even after discarding the top and bottom 5% of this distribution as possible outliers. The codon AAG in gene YAL038W, for example, occurs 36 times along this gene’s transcript. At the 196^th^ codon position AAG is translated in 363 ms, and at the 413^th^ position AAG is translated in 59 ms. Thus, the same codon in different contexts can be translated at very different speeds. Characterizing the distribution of mean times of translation of different occurrences of the same codon reveals a broad distribution (Fig 5A), whose coefficient of variation is often close to 0.5 (S1 File). This means that the standard deviation is half of the average translation time of a codon. This leads to the important finding that the average translation rate of a codon type is not representative of the rate at which most instances of that codon type are translated. These results are consistent with the findings that a large number of molecular factors determine codon translation rates *in vivo* [62], thus giving rise to a broad distribution of rates (Fig 5A, S9 Fig). These factors have been shown to cause a bias towards slower translation in the first 200 codons of many transcripts [23].

A molecular factor that has not been quantified in this study is ribosome queuing. Currently, the conventional Ribosome profiling protocol isolates only monosomes and the monosome-protected fragments are extracted and sequenced. However, should ribosomes queue along a transcript, disomes and trisomes are likely to be produced that are not accounted for in current datasets. Recent studies [14,63] have attempted to quantify the extent of ribosomal queuing but several challenges remain. One of the central challenges is to correctly identify the location of A-sites of ribosomes translating disome- and trisome-protected mRNA fragments. Current ribosome profiling datasets that include disomes have very sparse coverage, which limits the application of our method but more importantly suggests that the occurrence of disomes, and hence of queuing, may be rather rare under normal growth conditions. However, under stress conditions, ribosome queuing has the potential to become frequent for some genes and potentially decrease the accuracy of our method unless the disomes and trisomes fragments are included. As advances in ribosome profiling experiments are made to generate high coverage data and improve the A-site identification on disomes and trisomes, our method will be able to more accurately quantify the rates of translation elongation under non-standard growth conditions.

To determine the transcriptome-wide average elongation rate, Eq (7) was derived from the principles of mass flow and the continuity equation. Eq (7) accurately determines the average elongation rate in simulated data sets (Fig 2B). Applying Eq (7) to ribosome run-off experiments revealed that in mouse stem cells the average codon translation rate is 5.2 AA/s which is similar to the elongation rate measured in a previous study [26].

Measuring initiation rates using Eq (4) reproduced previously reported correlations (Fig 4A–4C), and also revealed a statistically significant correlation between the rate of translation-initiation and mRNA copy number (Fig 4E). This correlation indicates that genes with a higher mRNA copy number tend to have a higher translation efficiency, suggesting that transcriptional and translational regulation of gene expression can act synergistically to maximize the protein copy number of highly expressed genes.

Other methods have determined initiation rates by varying the initiation rate in simulations until the average ribosome density of a transcript matched the experimentally measured value [10,12,14,63]. These methods require knowledge of individual codon translation rates. Thus, small errors in these rates have the potential to accumulate and lead to large errors in the estimated initiation rates. This might contribute to conflicting conclusions reported in the literature. For example, three [10,14,16] out of four published methods, as well as our method, found a negative correlation between initiation rate and CDS length, whereas Gritsenko *et al*. [12] found no such correlation. In contrast to these methods, our method is based on a simple chemical kinetic equation that is easy to implement and does not require any detailed assumptions about codon translation rates. Apart from these simulation-based approaches, other methods [19,21,64,65] measure the protein synthesis rate, which represents a lower bound to the rate of translation-initiation (Eq (2)). One of these methods uses chemical kinetic modeling [21], but unlike Eq (4), this method requires a number of biophysical parameters, including the diffusion constant of tRNAs and ribosomes, and cell volume. The difference between the initiation rate and protein synthesis rate increases with increasing initiation rate [66]. Therefore, such methods could exhibit larger errors for transcripts that have higher translation-initiation rates, which are often found in highly expressed transcripts (Fig 4C and 4E).

A number of approaches have been developed to measure codon translation times including simulation based approaches [12,14] that extract rates by comparing the local distribution of ribosome profiling reads with simulated ribosome densities, others that optimize an objective function [13] or fit a normalized-footprint-count distribution of a codon to an empirical function [11], and yet others that measure relative codon translation times by quantifying the enrichment of ribosome read density using a variety of procedures [18,19]. In contrast, Eq (12) allows individual, absolute codon translation rates to be calculated directly from the ribosome profile along the transcript. Another distinction is that a number of these methods [11–13,19] assume that all occurrences of a codon across the transcriptome must be translated at the same rate. This assumption cannot be correct as it is known that non-local aspects of translation (such as mRNA structure) can influence the translation speed of individual codons. Eq (12) does not make this assumption, and therefore its extracted rates can better reflect the naturally occurring variation of codon translation times across a transcript.

The codon usage in a transcript, and associated translation rates, can affect various co- and post-translational processes involving nascent proteins [9]. Therefore, the accurate knowledge of codon translation times measured using Eq (12) will help provide a better quantitative understanding of how translation speed can impact the efficiency of co-translational processes, such as protein folding, chaperone binding, and numerous other processes involving the nascent protein. Coupled with molecular biology techniques that can knock out various genes and their functions in cells, Eq (12) provides the opportunity to quantitatively examine whether co-translational processes can cause translation speed changes.

Ribosome profiles have ill-quantified sequencing biases [27] that can potentially produce reads that are not proportional to the underlying number of ribosomes at a particular codon position. This could lead to errors in the extraction of translation rate parameters using our methods [67]. It has been demonstrated that using translational inhibitors like cycloheximide leads to distortion of ribosome profiles due to inefficient arrest of translation [39,68]. This was one of the primary reasons why initial studies using cycloheximide did not observe a correlation of codon translation rates and cognate tRNA concentration. While there is often a strong correlation between the total number of mapped reads per transcript between datasets from different studies, the correlation is often poor at the individual nucleotide level [69]. This “noise” at this resolution has been attributed to sparse read coverage [69], choice of ribonuclease for digestion [70], and the methods used to halt elongation in the ribosome profiling protocol [39,68]. Restricting our analyses to transcripts with high coverage contributes to more reproducible results, as can be seen by the high correlation between the two datasets used in this study (S10 Fig). Experimental improvements that minimize bias have been developed [16,34,70,71], such as using flash-freezing for arresting translation and utilizing short microRNA library generation techniques [72], but sequence-dependent biases can still exist, for example due to varying efficiencies of linker ligation [73]. As experimental techniques are improved to minimize bias, the accuracy of the rates extracted using our methods will also increase.

The absence of accurate translation rate parameters is an impediment to quantitatively modeling the process of translation. By measuring translation rate parameters using a chemical-kinetic framework, our methods can contribute to ongoing efforts [65,74] to understand how the sequence features of an mRNA molecule can regulate gene expression. More broadly, the approach we have taken in this study is to utilize ideas from chemistry and physics to analyze next-generation sequencing data; a branch of bioinformatics we refer to as physical bioinformatics. We expect that this physical-science-based approach will prove useful in understanding other large biological data sets concerning translation and compliment conventional computer science approaches to bioinformatics.

## Methods

### Simulated steady state and non-steady state ribosome profiling data

We carried out protein synthesis simulations using the inhomogeneous l-TASEP model [36,63,66,74,75]. In this model, with *ℓ* = 10 and the A-site of the ribosome located at the 6th codon within the ribosome-protected mRNA fragment, a new translation-initiation event stochastically occurs on transcript *i* with rate *α*(*i*) when the first six codons of the transcript are not occupied by another ribosome [15]. The ribosome then stochastically moves along the transcript from codon position *j* to *j* + 1 with rate *ω*(*j*, *i*) if no ribosome is present at the ${\left(j+l\right)}^{\text{th}}$ codon position. A ribosome stochastically terminates the translation process with rate *β* when its A-site encounters the stop codon. Note that our simulation model does not account for other processes such as ribosome recycling [44] and drop-off [76].

1,388 *S*. *cerevisiae* mRNA transcripts were selected to test our translation-initiation rate measurement method. They were chosen based on the filtering criteria that at least 95% of their codons have non-zero read density in the ribosome profiling data reported in Ref. [16]. The list of these genes are provided in S1 File. We used the translation-initiation rates reported in Ref. [10] in our simulations for 1,236 of the transcripts. The initiation rates for the other 152 transcripts were not reported in Ref. [10]. Therefore, we randomly assigned (with replacement) the initiation rate to those 152 transcripts from the same database. We used codon translation rates from Fluitt and Viljoen’s model for all 61 sense codons [38] and set the translation-termination rate to 35 *s*^−1^ [10]. We set l=10 codons in our simulations because it is the canonical mRNA fragment length that is protected by ribosomes against nucleotide digestion in ribosome profiling experiments [15].

We simulated the translation of these 1,388 *S*. *cerevisiae* mRNA sequences using the Gillespie’s algorithm [37] to generate the *in silico* ribosome profiling data. During the simulations, we recorded ribosome locations across the transcript every 100 steps, which we found minimized the time-correlation between successive saved snapshots. The codon positions of the ribosome’s A-site in each of these snapshots, summed over all snapshots, constituted the *in silico* generated ribosome profile for the transcript. We ran the simulations until the total number of *in silico* ribosome profiling reads were equal to the total number of reads aligned to the same transcript measured from experimental ribosome profiling data reported in Ref [16]. This allowed us to create a realistic level of statistical sampling in our *in silico* ribosome profiles. Each of the uncorrelated snapshots can be thought as a separate copy of the mRNA transcript. Thus, the total number of these snapshots were equal to the mRNA copy number in our *in silico* experiment which we used to calculate *ρ*(*j*, *i*)s.

Non-steady-state ribosome run-off experiments were simulated for 4,617 *S*. *cerevisiae* transcripts by using a Monte Carlo simulation method whose procedure is described in Refs. [77–79]. Like Gillespie’s algorithm [37], Monte Carlo simulation method also use exponentially distributed first passage times for each step in our simulation. We used Fluitt-Viljoen codon translation rates [38] and *in vivo* initiation rates reported in Ref. [10] to perform these non-steady-state simulations. These transcripts were chosen based on the filtering criteria reported in Ref. [26]. To perform the *in silico* run-off experiment described in Ref. [26], first we waited until the translation system achieved a steady-state and then set *α* = 0, which stopped translation-initiation in our simulations. However, we allowed ribosomes to continue elongation for a time Δ*t*, after which elongation was halted. Next, we recorded the ribosome A-site positions across the transcript that were defined as *in silico* ribosome profiling reads. We repeated this *in silico* experiment for the run-off times of Δ*t* = 5, 10, 15, 20, 25 and 30 seconds until the *in silico* average read per codon for each transcript became equal to the ribosome profiling reads reported in Ref. [16].

To test our method for measuring codon translation rates using Eq (12) we selected 85 transcripts that meet the filtering criteria (see sub-section *Selection of genes for codon translation rates* for details) from the experimental ribosome profiling data reported in Ref. [16]. We simulated protein synthesis on those 85 transcripts to generate *in silico* ribosome profiles. The number of *in silico* ribosome profiling reads for each of those 85 transcripts were equal to the experimental ribosome profiling reads reported in Ref. [16].

### *In silico* measurement of average protein synthesis and codon translation times

To calculate the translation-initiation rate (Eq (4)) and codon translation times (Eq (12)) from *in silico* ribosome profiles we need the average time a ribosome takes to synthesize a protein from a given transcript. We measured this quantity from our simulations by recording the time it takes a ribosome to traverse from the start codon to the stop codon in the transcript. The average synthesis time of a protein was then calculated from 10,000 individual ribosomes.

We also calculated the average synthesis time of a protein using a scaling relationship that uses the transcriptome-wide average codon translation time (S1 Text, Eq. (S10)). To calculate this quantity, we first computed the average codon translation time for each transcript by dividing the average protein synthesis time of a transcript by its CDS length. We then calculated the transcriptome-wide average codon translation time using the average codon translation time of each transcript.

Testing the accuracy of Eq (12) requires the real codon translation times which we measured by setting a separate clock at each codon position of a transcript in our simulations. These clocks measured the time difference between successive arrival and departure of a ribosome at each codon position. To calculate the average codon translation time at each codon position at least 10,000 such measurements were made.

### Calculation of the folding energy near the 5′ mRNA cap and estimation of other relevant parameters

We calculated the folding energy near the 5′ mRNA cap of *S*. *cerevisiae* transcripts to measure its correlation with *in vivo* translation-initiation rates calculated using Eq (4). The folding energy of the first 70 nucleotides after the 5′ mRNA cap correlates the most with the translation-initiation rate [16]. To identify the 5′ UTR sequences included in those 70 nucleotides, we used the database reported in Ref [80]. We calculated the folding energy of each segment by using the software RNAfold 2.0 [81].

We used the mRNA and protein copy numbers reported in Ref. [82] to measure their correlation with translation-initiation rates. The mRNA and protein copy number reported in this paper are the average copy numbers, which were each averaged using three independent studies [83–88].

### Analysis of ribosome profiling and RNA-Seq data

#### Datasets

To calculate the translation-initiation rates we applied our methods (Eq (4)) to *in vivo* ribosome profiling data published in Refs. [16] and [43] with NCBI accession numbers GSM1289257 and GSM1949551, respectively. The RNA-Seq data we used for Ref. [16] has NCBI accession number GSM1969535. For Ref. [43], we make public the RNA-Seq sample at GSM3242263 which we performed simultaneously with the ribosome profiling experiment. We chose these two data sets because they contain both ribosome profiling and RNA-Seq data of a sample, allowing us to calculate the average ribosome density of a transcript (S1 Text, Eq. (S9)). To calculate the codon translation rates, we apply our method to high-coverage ribosome profiling datasets of wild type *S*. *cerevisiae* reported in Refs. [43] and [53] with NCBI accession numbers GSM1949551 and GSM1495503, respectively. In our analysis, reads were preprocessed and mapped to sacCer3 reference genome as described in Ref. [43]. To maintain the accuracy of read assignment, transcripts in which multiple mapped reads constitute more than 0.1% of total reads mapping to the CDS region were not considered in the analysis. A-site positions in ribosome profiling reads were assigned according to the offset table generated using an Integer Programming algorithm which maximizes the reads between the second and stop codon of transcripts [89]. The offset table for *S*. *cerevisiae* is taken from Table 1 of ref. [89]. RPKM values were calculated for transcripts in RNA-Seq data by counting the reads whose 5′ ends were within the coding region of the transcript.

#### Selection of genes for translation-initiation rates

To apply our method (Eq (4)) for extracting translation initiation rates, we restrict our analysis to genes in which 95% of codon positions of a transcript have non-zero read density. 1,388 transcripts meet this criterion. This threshold reduces the statistical uncertainty in the estimation of codon position dependent ribosome density used in Eq (4).

#### Selection of genes for codon translation rates

To extract individual codon translation rates, we restrict our analysis to genes that have at least 3 reads at every codon position of the transcript. We find that 117 and 364 genes meet this criterion in the data set of Nissley *et al*. [43] and Williams *et al*. [53], respectively. This stringent requirement is necessary since Eq (12) would predict codons with zero reads to be translated in zero time. Reads at the start codon and the second codon have contributions from the translation initiation process; therefore, we ignored these codon positions in our calculations of translation time distributions and correlation with molecular factors. As stated before, transcripts containing multiple mapped reads greater than 0.1% of the total reads mapped to the transcript were discarded. Genes with overlap of coding sequence regions as well as those containing introns (which is less than 6% of *S*. *cerevisiae* genome) were not considered in the analysis to avoid overlap of ribosome profiles.

#### Preparation of RNA-Seq sample

200 mL of cells were grown in YPD to an OD_600 nm_ of 0.5, filtered (All-Glass Filter 90mm, Millipore), flash frozen in liquid nitrogen and lysed by mixer milling (2 min, 30 Hz, MM400, Retsch) with 600 μL of lysis buffer (20 mM Tris-HCl pH 8.0, 140 mM KCl, 6 mM MgCl2, 0.1% NP-40, 0.1 mg/ml cycloheximide, 1 mM PMSF, 2x protease inhibitors (Complete EDTA-free, Roche), 0.02 U/ml DNaseI (recombinant DNaseI, Roche), 20 mg/mL leupeptin, 20 mg/mL aprotinin, 10 mg/mL E-64, 40 mg/mL bestatin). Thawed cell lysates are cleared by centrifugation (20,000xg, 5 min, 4°C) and RNA extraction is performed as described in Ref. [90]. 10 μg of extracted RNA was depleted for rRNA using the kit RiboMinus, Yeast Module (Invitrogen) and fragmented for 30 min using the NEBNext Magnesium RNA Fragmentation Module (NEB). Deep sequencing libraries were prepared following the protocol described in Ref. [90] and sequenced on a HiSeq 2000 (Illumina).

#### Miscellaneous

(a) Since experimental measurements of 〈*T*(*i*)〉s are not available for *S*. *cerevisiae* we use Eq. (S10) to estimate 〈*T*(*i*)〉 with 〈*τ*^*A*^〉 = 200 ms, as reported in the literature [40,41]. (b) *In vivo* ribosome profiling data for the mouse stem cells were processed using the method described in the original paper [26]. (c) The measures for tRNA abundance based on gene copy number and RNA-Seq measurements were obtained from Table S2 of Ref. [16]. (d) Transcript leader (or 5′ UTR) sequences were obtained from Ref. [80] and upstream AUG were identified in these sequences for the transcripts for which we calculate the translation initiation rates. (e) For Kozak sequence analysis, 12 nt sequence was identified around the start codon starting from -6 with respect to Adenine of start codon (position +1) to +6. The consensus Kozak sequence for *S*. *cerevisiae* is (A/T)A(A/C)A(A/C)AATGTC(T/C) [49]. For the 12 nt sequence for every transcript, a similarity score is calculated based on its match with the Kozak sequence. The score ranges from 1 to 10 in the order of its increasing similarity with Kozak sequence. If all the 9 nt around the start codon are same as the Kozak sequence and the start codon (scored as 1), the score will be 9+1 = 10. If only start codon is same while all other 9 nt are different, the score is just 1. If start codon is same and only 4 nt positions are same, then the score will be 4+1 = 5. To determine the effect of Kozak sequence, two subsets of transcripts were created, the first with transcripts having context around start codon closer to Kozak sequence (score > 7) and the second subset for transcripts with context farther away from Kozak sequence (score < 5). Mann-Whitney U test was used to determine the statistical significance of the difference between the translation-initiation rate distributions of the two subsets of genes.

### Assignment of mRNA secondary structure

Both DMS and PARS data provide information about base-paired nucleotides within an mRNA molecule. We considered a codon to be structured if at least two of its three nucleotides were base-paired or one nucleotide was base-paired and the structure information for the other two nucleotides was not available.

DMS data for *S*. *cerevisiae* were downloaded from GEO database with accession number GSE45803 [58]. The reads from all *in vivo* replicates were pooled together and then aligned to the ribosomal RNA sequences using Bowtie 2 (v2.2.3) [91]. The reads which did not align to the ribosomal RNA sequences were then aligned to the *Saccharomyces cerevisiae* assembly R64-2-1 (UCSC: sacCer3) using Tophat (v2.0.13) [92]. In our analysis, A and C nucleotides were considered base-paired when the DMS signal was below the threshold of 0.2 and considered unstructured if the DMS signal was greater than 0.5. A and C nucleotides with DMS signal between 0.2 and 0.5 are considered ambiguous and classified together with U and G nucleotides, which do not react with DMS. Codons involving such nucleotides were not considered in our analysis.

PARS data were downloaded from genie.weizmann.ac.il/pubs/PARS10 with PARS scores available for all transcripts, except YDR461W and YNL145W, which were excluded from our analysis. Nucleotides with a PARS score greater than 0 were considered base-paired [59].

## Supporting information

S1 Text Detailed derivations of rates discussed in Theory section. (PDF)

S1 Fig Normalized ribosome read density calculated from run-off experiments decreases linearly as a function of time. (**A**) The normalized ribosome read density in *S*. *cerevisiae* using *in silico* run-off experiment data in the first 201, 250, 300, 350 and 400 codons are plotted as a function of time. (**B**) The normalized ribosome read density in mouse stem cells (Ref. [26]) are plotted as a function of time in the first 800, 850, 900, 950 and 1000 codons. Lines are to guide the eye.
(TIFF)

S2 Fig Eq (4) accurately determines the translation-initiation rate from simulated *S*. *cerevisiae* ribosome profiles where the translation rates of the first 100 codons were artificially decreased. (**A**) Translation-initiation rates determined by applying Eq (4) to simulated ribosome profiling data are plotted against the actual initiation rates used in the simulations. These initiation rates were calculated using Eq. (S10) for the protein synthesis times. (**B**) Same as (**A**) but the average protein synthesis times were measured from our simulations of the translation process. The solid lines in (**A**) and (**B**) are the lines of the best fit.
(TIFF)

S3 Fig Comparison between translation-initiation rates measured from two independent data sets. *α*_1_ and *α*_2_ are the translation-initiation rate calculated by Eq (4) using the ribosome profiling and RNA-Seq data reported in Nissley *et al*. [43] and Weinberg *et al*. [16], respectively. Polysome profiling data reported in Mackay *et al*. [42] and Arava *et al*. [29] were used in (**A**) and (**B**), respectively.
(TIFF)

S4 Fig Comparison between translation-initiation rates measured from Eq (4) with those of Dao Duc and Song. [14]. *In vivo* initiation rates calculated by Eq (4) using the ribosome profiling and RNA-seq data from Weinberg *et al*. [16] were compared with the ones reported in Dao Duc and Song [14] in (**A**) and (**C**); *In vivo* initiation rates calculated by Eq (4) using the ribosome profiling and RNA-seq data from Nissley *et al*. [43] were compared with the ones reported in Dao Duc and Song [14] in (**B**) and (**D**). Polysome profiling data reported in Mackay *et al*. [42] were used to calculate *in vivo* initiation rates in (**A**) and (**B**); Polysome profiling data reported in Arava *et al*. [29] were used to calculate *in vivo* nitiation rates in (**C**) and (**D**).
(TIFF)

S5 Fig Translation-initiation rates measured from data sets taken from Refs. [16] and [29] reproduce previously reported correlations. *In vivo* translation-initiation rates of *S*. *cerevisiae* transcripts are plotted against the inverse of their CDS length, folding energy of mRNA molecule near the 5′ cap and protein copy number in (**A**), (**B**) and (**C**), respectively. (**D**) The copy number of *S*. *cerevisiae* proteins are plotted as a function of the product of the initiation rate of transcripts that encode them and that transcript’s copy number in a cell. (**E**) mRNA copy number is plotted against the translation-initiation rate.
(TIFF)

S6 Fig Translation-initiation rates measured from data sets taken from Refs. [43] and [42] reproduce previously reported correlation. *In vivo* translation-initiation rates of *S*. *cerevisiae* transcripts are plotted against the inverse of their CDS length, folding energy of mRNA molecule near the 5′ cap and protein copy number in (**A**), (**B**) and (**C**), respectively. (**D**) The copy number of *S*. *cerevisiae* proteins are plotted as a function of the product of the initiation rate of transcripts that encode them and that transcript’s copy number in a cell. (**E**) mRNA copy number is plotted against the translation-initiation rate.
(TIFF)

S7 Fig Translation-initiation rates calculated from data sets taken from Refs. [43] and [29] reproduce previously reported correlations. *In vivo* translation-initiation rates of *S*. *cerevisiae* transcripts are plotted against the inverse of their CDS length, folding energy of mRNA molecule near the 5′ cap and protein copy number in (**A**), (**B**) and (**C**), respectively. (**D**) The copy number of *S*. *cerevisiae* proteins are plotted as a function of the product of the initiation rate of transcripts that encode them and that transcript’s copy number in a cell. (**E**) mRNA copy number is plotted against the translation-initiation rate.
(TIFF)

S8 Fig Comparison of the properties of the 117- and 364-transcript data sets from Refs. [43] and [53], respectively, to the entire *S*. *cerevisiae* transcriptome. Probability distributions of CDS length and percent GC content from the data set of 117-transcripts from Ref. [43] (green) and from the entire transcriptome (blue) are plotted in (**A**) and (**B**), respectively. (**C**) Scatter plot of the codon usage in the whole genome versus the 117-transcript data set from Ref. [43]. (**D**), (**E**) and (**F**) are the same as (**A**), (**B**) and (**C**), respectively, except 364-transcripts from Ref. [53] is used.
(TIFF)

S9 Fig Translation time distributions for the 64 codon types. **(A)** The translation time distributions for each codon type is shown for the dataset of Nissley *et al*. [43]. The distribution is shown ignoring the extreme 5^th^ percentiles at both ends of the distribution. The codons are sorted based on the medians of their translation time distributions. There are only three instances of CGG and one instance of CGA in our gene subset and hence their boxplot is not noticeable. **(B)** Same as (**A**) but for the dataset of Williams *et al*. [53]. The sorting is the same as in (**A**).
(TIFF)

S10 Fig Codon translation rates are highly correlated across datasets and with rates from method of Dao Duc and Song. **(A)** The medians of the translation time distributions of the 64 codon types are highly correlated between the datasets of Nissley *et al*. [43] and Williams *et al*. [53]. **(B)** The standard deviations of these translation time distributions are also highly correlated for the two datasets indicating that the variability of translation times is reproducible across datasets. **(C)** The codon translation rates obtained using Eq (12) for the dataset from Weinberg *et al*. [16] is correlated with codon translation rates inferred in the study of Dao Duc and Song [14] on the same dataset.
(TIFF)

S11 Fig Molecular factors shaping the variability of individual codon translation rates in the dataset from Ref [53]. **(A-B)** Median translation times of codon types are negatively correlated with cognate tRNA abundance estimated by **(A)** gene copy number and **(B)** RNA-Seq gene expression. **(C)** Probability distribution of translation time of codons in the A-site either when a proline is present in the P-site (green) or when a proline is not present in the P-site (blue). **(D-E)** Percentage difference in median translation times when mRNA structure is present relative to when it is not present as a function of codon position after the A-site. Grey bars indicate results that are not statistically significant. Error bars are the 95% C.I. calculated using 10^4^ bootstrap cycles; significance is assessed using the Mann-Whitney U test corrected with the Benjamini Hochberg FDR method for multiple-hypothesis correction. mRNA structure information used in (**D**) and (**E**) were taken from *in vivo* DMS and *in vitro* PARS data, respectively. **(F)** Scatter plot of the median translation times of pairs of codon types that are decoded by the same tRNA molecule. The red line is the identity line. The list of tRNA molecules and which codon they decode were taken from Ref. [54]. Error bars are standard error about the median calculated with 10^4^ bootstrap cycles.
(TIFF)

S12 Fig Average ribosome density on a transcript as a function of translation efficiency. Translation efficiency in (**A**) and (**B**) are calculated using the ribosome profiling and RNA-Seq data reported in Ref. [16]; Translation efficiency in (**C**) and (**D**) are calculated using ribosome profiling and RNA-Seq data reported in Ref. [43]. Ribosome density used in (**A**) and (**C**) are from the polysome profiling data reported in Ref. [42] whereas the ribosome density in (**B**) and (**D**) are provided by Ref. [29]. The solid line in all these figures represent the best fit of *y* = *ξx* line.
(TIFF)

S1 Table Transcripts containing at least one upstream AUG (uAUG) have lower median translation-initiation rates. For all possible combinations of ribosome profiling and RNA-Seq data [16,43] and polysome profiling data [29,42], we see a consistent result that the median translation-initiation rate is lower for transcripts with at least one uAUG. The result is not statistically significant for combination of Refs. [43] and [42] (p-value = 0.052).
(PDF)

S2 Table Transcripts with sequence context around start codon similar to Kozak sequence have higher translation-initiation rates. For all possible combinations of ribosome profiling and RNA-Seq data [16,43] and polysome profiling data [29,42], we see a consistent result that the median translation-initiation rate is higher for transcripts with sequence context similar to Kozak sequence (see Methods for details). The result for combination of Refs [43] and [29] is however not statistically significant (p-value = 0.065).
(PDF)

S1 File Initiation rates for genes discussed in the study as well as statistics for codon translation times for 64 codon types. (XLSX)

## References

[1] Chowdhury D. Stochastic mechano-chemical kinetics of molecular motors: A multidisciplinary enterprise from a physicist’s perspective. Phys Rep. 2013;529: 1–197. 10.1016/j.physrep.2013.03.005

[2] Marshall RA, Aitken CE, Dorywalska M, Puglisi JD. Translation at the single-molecule level. Annu Rev Biochem. 2008;77: 177–203. 10.1146/annurev.biochem.77.070606.101431 18518820

[3] Sharma AK, Chowdhury D. Template-directed biopolymerization:Tape-copying turing machines. Biophys Rev Lett. 2012;7: 135–175. 10.1142/S1793048012300083

[4] Jackson RJ, Hellen CUT, Pestova T V. The mechanism of eukaryotic translation initiation and principles of its regulation. Nat Rev Mol Cell Biol. 2010;11: 113–127. 10.1038/nrm2838 20094052 PMC4461372

[5] Hinnebusch AG. The Scanning Mechanism of Eukaryotic Translation Initiation. Annu Rev Biochem. 2014;83: 779–812. 10.1146/annurev-biochem-060713-035802 24499181

[6] Espah Borujeni A, Salis HM. Translation Initiation is Controlled by RNA Folding Kinetics via a Ribosome Drafting Mechanism. J Am Chem Soc. 2016;138: 7016–7023. 10.1021/jacs.6b01453 27199273

[7] Spriggs KA, Bushell M, Willis AE. Translational Regulation of Gene Expression during Conditions of Cell Stress. Mol Cell. 2010;40: 228–237. 10.1016/j.molcel.2010.09.028 20965418

[8] Kervestin S, Amrani N. Translational regulation of gene expression. Genome Biol. 2004;5: 359 10.1186/gb-2004-5-12-359 15575982 PMC545794

[9] Sharma AK, O’Brien EP. Non-equilibrium coupling of protein structure and function to translation–elongation kinetics. Curr Opin Struct Biol. 2018;49: 94–103. 10.1016/j.sbi.2018.01.005 29414517

[10] Ciandrini L, Stansfield I, Romano MC. Ribosome Traffic on mRNAs Maps to Gene Ontology: Genome-wide Quantification of Translation Initiation Rates and Polysome Size Regulation. PLoS Comput Biol. 2013;9: e1002866 10.1371/journal.pcbi.1002866 23382661 PMC3561044

[11] Dana A, Tuller T. Mean of the Typical Decoding Rates: A New Translation Efficiency Index Based on the Analysis of Ribosome Profiling Data. G3-Genes Genomes Genet. 2015;5: 73–80. 10.1534/g3.114.015099 25452418 PMC4291471

[12] Gritsenko AA, Hulsman M, Reinders MJT, de Ridder D. Unbiased Quantitative Models of Protein Translation Derived from Ribosome Profiling Data. PLOS Comput Biol. 2015;11: e1004336 10.1371/journal.pcbi.1004336 26275099 PMC4537299

[13] Pop C, Rouskin S, Ingolia NT, Han L, Phizicky EM, Weissman JS, et al Causal signals between codon bias, mRNA structure, and the efficiency of translation and elongation. Mol Syst Biol. 2014;10: 770 10.15252/msb.20145524 25538139 PMC4300493

[14] Dao Duc K, Song YS. The impact of ribosomal interference, codon usage, and exit tunnel interactions on translation elongation rate variation. PLoS Genet. 2018;14: e1001508 10.1371/journal.pgen.1007166 29337993 PMC5786338

[15] Ingolia NT, Ghaemmaghami S, Newman JRS, Weissman JS. Genome-wide analysis in vivo of translation with nucleotide resolution using ribosome profiling. Science. 2009;324: 218–223. 10.1126/science.1168978 19213877 PMC2746483

[16] Weinberg DE, Shah P, Eichhorn SW, Hussmann JA, Plotkin JB, Bartel DP. Improved Ribosome-Footprint and mRNA Measurements Provide Insights into Dynamics and Regulation of Yeast Translation. Cell Rep. 2016;14: 1787–1799. 10.1016/j.celrep.2016.01.043 26876183 PMC4767672

[17] Artieri CG, Fraser HB. Accounting for biases in riboprofiling data indicates a major role for proline in stalling translation. Genome Res. 2014;24: 2011–2021. 10.1101/gr.175893.114 25294246 PMC4248317

[18] Gardin J, Yeasmin R, Yurovsky A, Cai Y, Skiena S, Futcher B. Measurement of average decoding rates of the 61 sense codons in vivo. Elife. 2014;3: e03735 10.7554/eLife.03735 25347064 PMC4371865

[19] Qian W, Yang JR, Pearson NM, Maclean C, Zhang J. Balanced codon usage optimizes eukaryotic translational efficiency. PLoS Genet. 2012;8: e1002603 10.1371/journal.pgen.1002603 22479199 PMC3315465

[20] Charneski CA, Hurst LD. Positively Charged Residues Are the Major Determinants of Ribosomal Velocity. PLoS Biol. 2013;11: e1001508 10.1371/journal.pbio.1001508 23554576 PMC3595205

[21] Shah P, Ding Y, Niemczyk M, Kudla G, Plotkin JB. Rate-limiting steps in yeast protein translation. Cell. 2013;153: 1589–601. 10.1016/j.cell.2013.05.049 23791185 PMC3694300

[22] Dana A, Tuller T. The effect of tRNA levels on decoding times of mRNA codons. Nucleic Acids Res. 2014;42: 9171–9181. 10.1093/nar/gku646 25056313 PMC4132755

[23] Tuller T, Veksler-Lublinsky I, Gazit N, Kupiec M, Ruppin E, Ziv-Ukelson M. Composite effects of gene determinants on the translation speed and density of ribosomes. Genome Biol. 2011;12 10.1186/gb-2011-12-11-r110 22050731 PMC3334596

[24] Tuller T, Waldman YY, Kupiec M, Ruppin E. Translation efficiency is determined by both codon bias and folding energy. Proc Natl Acad Sci. 2010;107: 3645–3650. 10.1073/pnas.0909910107 20133581 PMC2840511

[25] Requião RD, de Souza HJA, Rossetto S, Domitrovic T, Palhano FL. Increased ribosome density associated to positively charged residues is evident in ribosome profiling experiments performed in the absence of translation inhibitors. RNA Biol. 2016;13: 561–568. 10.1080/15476286.2016.1172755 27064519 PMC4962802

[26] Ingolia NT, Lareau LF, Weissman JS. Ribosome profiling of mouse embryonic stem cells reveals the complexity and dynamics of mammalian proteomes. Cell. 2011;147: 789–802. 10.1016/j.cell.2011.10.002 22056041 PMC3225288

[27] Dana A, Tuller T. Determinants of Translation Elongation Speed and Ribosomal Profiling Biases in Mouse Embryonic Stem Cells. PLoS Comput Biol. 2012;8 10.1371/journal.pcbi.1002755 23133360 PMC3486846

[28] Szavits-Nossan J, Ciandrini L, Romano MC. Deciphering mRNA Sequence Determinants of Protein Production Rate. Phys Rev Lett. American Physical Society; 2018;120: 128101 10.1103/PhysRevLett.120.128101 29694095

[29] Arava Y, Wang Y, Storey JD, Liu CL, Brown PO, Herschlag D. Genome-wide analysis of mRNA translation profiles in Saccharomyces cerevisiae. Proc Natl Acad Sci U S A. 2003;100: 3889–3894. 10.1073/pnas.0635171100 12660367 PMC153018

[30] MacDonald CT, Gibbs JH, Pipkin AC. Kinetics of biopolymerization on nucleic acid templates. Biopolymers. 1968;6: 1–25. 10.1002/bip.1968.360060102 5641411

[31] Sharma AK, Ahmed N, O’Brien EP. Determinants of translation speed are randomly distributed across transcripts resulting in a universal scaling of protein synthesis times. Phys Rev E. 2018;97: 22409 10.1103/PhysRevE.97.022409 29548178

[32] Oh E, Becker AH, Sandikci A, Huber D, Chaba R, Gloge F, et al Selective ribosome profiling reveals the cotranslational chaperone action of trigger factor in vivo. Cell. 2011;147: 1295–1308. 10.1016/j.cell.2011.10.044 22153074 PMC3277850

[33] Griffiths DJ. An Introduction to Electrodynamics. Prentice Hall 1999.

[34] Ingolia NT, Brar GA, Rouskin S, McGeachy AM, Weissman JS. The ribosome profiling strategy for monitoring translation in vivo by deep sequencing of ribosome-protected mRNA fragments. Nat Protoc. 2012;7: 1534–1550. 10.1038/nprot.2012.086 22836135 PMC3535016

[35] Dao Duc K, Saleem ZH, Song YS. Theoretical analysis of the distribution of isolated particles in totally asymmetric exclusion processes: Application to mRNA translation rate estimation. Phys Rev E. 2018;97: 12106 10.1103/PhysRevE.97.012106 29448386

[36] Chou T, Mallick K, Zia RKP. Non-equilibrium statistical mechanics: from a paradigmatic model to biological transport. Reports Prog Phys. 2011;74: 116601.

[37] Gillesple DT. Exact Stochastic Simulation of couple chemical reactions. J Phys Chem. 1977;81: 2340–2361.

[38] Fluitt A, Pienaar E, Viljoen H. Ribosome kinetics and aa-tRNA competition determine rate and fidelity of peptide synthesis. Comput Biol Chem. 2007;31: 335–346. 10.1016/j.compbiolchem.2007.07.003 17897886 PMC2727733

[39] Hussmann JA, Patchett S, Johnson A, Sawyer S, Press WH. Understanding Biases in Ribosome Profiling Experiments Reveals Signatures of Translation Dynamics in Yeast. PLoS Genet. 2015;11: e1005732 10.1371/journal.pgen.1005732 26656907 PMC4684354

[40] Bonven B, Gulløv K. Peptide chain elongation rate and ribosomal activity in Saccharomyces cerevisiae as a function of the growth rate. Mol Gen Genet. 1979;170: 225–30. 10.1007/BF00337800 372763

[41] Karpinets T V, Greenwood DJ, Sams CE, Ammons JT. RNA:protein ratio of the unicellular organism as a characteristic of phosphorous and nitrogen stoichiometry and of the cellular requirement of ribosomes for protein synthesis. BMC Biol. 2006;4: 30 10.1186/1741-7007-4-30 16953894 PMC1574349

[42] MacKay VL, Li X, Flory MR, Turcott E, Law GL, Serikawa KA, et al Gene Expression Analyzed by High-resolution State Array Analysis and Quantitative Proteomics. Mol Cell Proteomics. 2004;3: 478–489. 10.1074/mcp.M300129-MCP200 14766929

[43] Nissley DA, Sharma AK, Ahmed N, Friedrich UA, Kramer G, Bukau B, et al Accurate prediction of cellular co-translational folding indicates proteins can switch from post- to co-translational folding. Nat Commun. 2016;7: 10341 10.1038/ncomms10341 26887592 PMC4759629

[44] Fernandes LD, de Moura A, Ciandrini L. Gene length as a regulator for ribosome recruitment and protein synthesis: theoretical insights. Sci Rep. 2017;7: 17409 10.1101/105296 29234048 PMC5727216

[45] Salis HM, Mirsky EA, Voigt CA. Automated design of synthetic ribosome binding sites to control protein expression. Nat Biotechnol. 2009;27: 946–50. 10.1038/nbt.1568 19801975 PMC2782888

[46] Kudla G, Murray AW, Tollervey D, Plotkin JB. Coding-Sequence Determinants of Gene Expression in Escherichia coli. Science. 2009;324: 255–258. 10.1126/science.1170160 19359587 PMC3902468

[47] Kochetov A V. Alternative translation start sites and hidden coding potential of eukaryotic mRNAs. BioEssays. 2008;30: 683–691. 10.1002/bies.20771 18536038

[48] Hood HM, Neafsey DE, Galagan J, Sachs MS. Evolutionary Roles of Upstream Open Reading Frames in Mediating Gene Regulation in Fungi. Annu Rev Microbiol. 2009;63: 385–409. 10.1146/annurev.micro.62.081307.162835 19514854

[49] Hamilton R, Watanabe CK, De Boer H a, Development P, Francisco SS. Compilation and comparison of the sequence context around the AUG start codons in Saccharomyces cerevisiae mRNAs. Nucleic Acids Res. 1987;15: 3581–3593. 10.1093/nar/15.8.3581 3554144 PMC340751

[50] Dvir S, Velten L, Sharon E, Zeevi D, Carey LB, Weinberger A, et al Deciphering the rules by which 5 ‘-UTR sequences affect protein expression in yeast. Proc Natl Acad Sci U S A. 2013;110: E2792–E2801. 10.1073/pnas.1222534110 23832786 PMC3725075

[51] Reeve B, Hargest T, Gilbert C, Ellis T. Predicting translation initiation rates for designing synthetic biology. Front Bioeng Biotechnol. 2014;2: 1 10.3389/fbioe.2014.00001 25152877 PMC4126478

[52] Csárdi G, Franks A, Choi DS, Airoldi EM, Drummond DA. Accounting for Experimental Noise Reveals That mRNA Levels, Amplified by Post- Transcriptional Processes, Largely Determine Steady-State Protein Levels in Yeast. PLoS Genet. 2015;11: e1005206 10.1371/journal.pgen.1005206 25950722 PMC4423881

[53] Williams CC, Jan CH, Weissman JS. Targeting and plasticity of mitochondrial proteins revealed by proximity-specific ribosome profiling. Science. 2014;346: 748–751. 10.1126/science.1257522 25378625 PMC4263316

[54] Cannarrozzi G, Schraudolph NN, Faty M, von Rohr P, Friberg MT, Roth AC, et al A role for codon order in translation dynamics. Cell. 2010;141: 355–367. 10.1016/j.cell.2010.02.036 20403329

[55] Pavlov MY, Watts RE, Tan Z, Cornish VW, Ehrenberg M, Forster AC. Slow peptide bond formation by proline and other N-alkylamino acids in translation. Proc Natl Acad Sci U S A. 2009;106: 50–54. 10.1073/pnas.0809211106 19104062 PMC2629218

[56] Der Wen J, Lancaster L, Hodges C, Zeri AC, Yoshimura SH, Noller HF, et al Following translation by single ribosomes one codon at a time. Nature. 2008;452: 598–603. 10.1038/nature06716 18327250 PMC2556548

[57] Qu X, Wen J-D, Lancaster L, Noller HF, Bustamante C, Tinoco I. The ribosome uses two active mechanisms to unwind messenger RNA during translation. Nature. Nature Publishing Group; 2011;475: 118–121. 10.1038/nature10126 21734708 PMC4170678

[58] Rouskin S, Zubradt M, Washietl S, Kellis M, Weissman JS. Genome-wide probing of RNA structure reveals active unfolding of mRNA structures in vivo. Nature. 2014;505: 701–705. 10.1038/nature12894 24336214 PMC3966492

[59] Kertesz M, Wan Y, Mazor E, Rinn JL, Nutter RC, Chang HY, et al Genome-wide measurement of RNA secondary structure in yeast. Nature. 2010;467: 103–107. 10.1038/nature09322 20811459 PMC3847670

[60] Sorensen MA, Pedersen S. Absolute in vivo translation rates of individual codons in Escherichia coli. The two glutamic acid codons GAA and GAG are translated with a threefold difference in rate. J Mol Biol. 1991;222: 265–280. 10.1016/0022-2836(91)90211-N 1960727

[61] Stadler M, Fire A. Wobble base-pairing slows in vivo translation elongation in metazoans. RNA. 2011;17: 2063–2073. 10.1261/rna.02890211 22045228 PMC3222120

[62] Brar GA. Beyond the Triplet Code: Context Cues Transform Translation. Cell. 2016 pp. 1681–1692. 10.1016/j.cell.2016.09.022 27984720 PMC6140790

[63] Diament A, Feldman A, Schochet E, Kupiec M, Arava Y, Tuller T. The extent of ribosome queuing in budding yeast. PLoS Comput Biol. 2018;14: e1005951 10.1371/journal.pcbi.1005951 29377894 PMC5805374

[64] Li GW, Burkhardt D, Gross C, Weissman JS. Quantifying absolute protein synthesis rates reveals principles underlying allocation of cellular resources. Cell. 2014;157: 624–635. 10.1016/j.cell.2014.02.033 24766808 PMC4006352

[65] Schwanhäusser B, Busse D, Li N, Dittmar G, Schuchhardt J, Wolf J, et al Global quantification of mammalian gene expression control. Nature. 2011;473: 337–342. 10.1038/nature10098 21593866

[66] Shaw LB, Zia RKP, Lee KH. Totally asymmetric exclusion process with extended objects: A model for protein synthesis. Phys Rev E. 2003;68: 021910 10.1103/PhysRevE.68.021910 14525009

[67] Ingolia NT. Ribosome Footprint Profiling of Translation throughout the Genome. Cell. 2016;165: 22–33. 10.1016/j.cell.2016.02.066 27015305 PMC4917602

[68] Gerashchenko M V., Gladyshev VN. Translation inhibitors cause abnormalities in ribosome profiling experiments. Nucleic Acids Res. 2014;42 10.1093/nar/gku671 25056308 PMC4176156

[69] Diament A, Tuller T. Estimation of ribosome profiling performance and reproducibility at various levels of resolution. Biol Direct. 2016;11: 24 10.1186/s13062-016-0127-4 27160013 PMC4862193

[70] Gerashchenko M V., Gladyshev VN. Ribonuclease selection for ribosome profiling. Nucleic Acids Res. 2017;45: e6 10.1093/nar/gkw822 27638886 PMC5314788

[71] Lecanda A, Nilges BS, Sharma P, Nedialkova DD, Schwarz J, Vaquerizas JM, et al Dual randomization of oligonucleotides to reduce the bias in ribosome-profiling libraries. Methods. 2016;107: 89–97. 10.1016/j.ymeth.2016.07.011 27450428 PMC5024760

[72] Sébastien P, Mariana L-Q, Thomas T. Cloning of Small RNA Molecules. Curr Protoc Mol Biol. 2005;72: 26.4.1–26.4.18. 10.1002/0471142727.mb2604s72 18265364

[73] Levin JZ, Yassour M, Adiconis X, Nusbaum C, Thompson DA, Friedman N, et al Comprehensive comparative analysis of strand-specific RNA sequencing methods. Nat Methods. 2010;7: 709–715. 10.1038/nmeth.1491 20711195 PMC3005310

[74] Zur H, Tuller T. Predictive biophysical modeling and understanding of the dynamics of mRNA translation and its evolution. Nucleic Acids Res. 2016;44: 9031–9049. 10.1093/nar/gkw764 27591251 PMC5100582

[75] Lakatos G, Chou T. Totally asymmetric exclusion processes with particles of arbitrary size. 2003;36: 2027–2041.

[76] Bonnin P, Kern N, Young NT, Stansfield I, Romano MC. Novel mRNA-specific effects of ribosome drop-off on translation rate and polysome profile. PLoS Comput Biol. 2017; 10.1371/journal.pcbi.1005555 28558053 PMC5469512

[77] Zia RKP, Dong JJ, Schmittmann B. Modeling Translation in Protein Synthesis with TASEP: A Tutorial and Recent Developments. J Stat Phys. 2011;144: 405–428. 10.1007/s10955-011-0183-1

[78] Sharma AK, Chowdhury D. Distribution of dwell times of a ribosome: effects of infidelity, kinetic proofreading and ribosome crowding. Phys Biol. 2011;8: 26005 10.1088/1478-3975/8/2/026005 21263169

[79] Sharma AK, Chowdhury D. Stochastic theory of protein synthesis and polysome: Ribosome profile on a single mRNA transcript. J Theor Biol. 2011;289: 36–46. 10.1016/j.jtbi.2011.08.023 21888920

[80] Arribere JA, Gilbert W V. Roles for transcript leaders in translation and mRNA decay revealed by transcript leader sequencing. Genome Res. 2013;23: 977–987. 10.1101/gr.150342.112 23580730 PMC3668365

[81] Lorenz R, Bernhart SH, Höner zu Siederdissen C, Tafer H, Flamm C, Stadler PF, et al ViennaRNA Package 2.0. Algorithms Mol Biol. 2011;6: 26 10.1186/1748-7188-6-26 22115189 PMC3319429

[82] Lu P, Vogel C, Wang R, Yao X, Marcotte EM. Absolute protein expression profiling estimates the relative contributions of transcriptional and translational regulation. Nat Biotechnol. 2007;25: 117–124. 10.1038/nbt1270 17187058

[83] Velculescu VE, Zhang L, Zhou W, Vogelstein J, Basrai MA, Bassett DE, et al Characterization of the yeast transcriptome. Cell. 1997;88: 243–251. 10.1016/S0092-8674(00)81845-0 9008165

[84] Holstege FCP, Jennings EG, Wyrick JJ, Lee TI, Hengartner CJ, Green MR, et al Dissecting the regulatory circuitry of a eukaryotic genome. Cell. 1998;95: 717–728. 10.1016/S0092-8674(00)81641-4 9845373

[85] Wang Y, Liu CL, Storey JD, Tibshirani RJ, Herschlag D, Brown PO. Precision and functional specificity in mRNA decay. Proc Natl Acad Sci. 2002;99: 5860–5865. 10.1073/pnas.092538799 11972065 PMC122867

[86] Newman JRS, Ghaemmaghami S, Ihmels J, Breslow DK, Noble M, DeRisi JL, et al Single-cell proteomic analysis of S. cerevisiae reveals the architecture of biological noise. Nature. 2006;441: 840–846. 10.1038/nature04785 16699522

[87] Ghaemmaghami S, Huh W-K, Bower K, Howson RW, Belle A, Dephoure N, et al Global analysis of protein expression in yeast. Nature. 2003;425: 737–741. 10.1038/nature02046 14562106

[88] Futcher B, Latter GI, Monardo P, McLaughlin CS, Garrels JI. A Sampling of the Yeast Proteome. Mol Cell Biol. 1999;19: 7357–7368. 10.1128/mcb.19.11.7357 10523624 PMC84729

[89] Ahmed N, Sormanni P, Ciryam P, Vendruscolo M, Dobson CM, O’Brien EP. Identifying A- and P-site locations on ribosome-protected mRNA fragments using Integer Programming. Sci Reports. 2019;9: 6256 10.1038/s41598-019-42348-x 31000737 PMC6472398

[90] Döring K, Ahmed N, Riemer T, Suresh HG, Vainshtein Y, Habich M, et al Profiling Ssb-Nascent Chain Interactions Reveals Principles of Hsp70-Assisted Folding. Cell. 2017;170: 298–311.e20. 10.1016/j.cell.2017.06.038 28708998 PMC7343536

[91] Langmead B, Salzberg SL. Fast gapped-read alignment with Bowtie 2. Nat Methods. 2012;9: 357–359. 10.1038/nmeth.1923 22388286 PMC3322381

[92] Kim D, Pertea G, Trapnell C, Pimentel H, Kelley R, Salzberg SL. TopHat2: accurate alignment of transcriptomes in the presence of insertions, deletions and gene fusions. Genome Biol. 2013;14: R36 10.1186/gb-2013-14-4-r36 23618408 PMC4053844

