# Supplementary material for: A chemical kinetic basis for measuring translation initiation and elongation rates from ribosome profiling data
Source: PLoS Comput Biol. 2019 May 23;15(5):e1007070. doi: 10.1371/journal.pcbi.1007070 (PMC6559674; doi:10.1371/journal.pcbi.1007070)
Supplement: S1 Text — (PDF) [file pcbi.1007070.s001.pdf]

A Chemical Kinetic Basis for Measuring Translation Initiation and Elongation Rates from Ribosome Profiling data

Ajeet K. Sharma[1, ‡, #], Pietro Sormanni[2, ‡], Nabeel Ahmed[3, ‡], Prajwal Ciryam[2, &], Ulrike A. Friedrich[4, 5], Günter Kramer[4, 5] and Edward P. O'Brien[1, 3, 6, *]

[1] Department of Chemistry, Pennsylvania State University, University Park, PA 16802, USA

[2] Center for Misfolding Diseases, Department of Chemistry, University of Cambridge, Cambridge, CB2 1EW, UK

[3] Bioinformatics and Genomics Graduate Program, The Huck Institutes of the Life Sciences, Pennsylvania State University, University Park, PA 16802, USA

[4] Center for Molecular Biology of the Heidelberg University (ZMBH), DKFZ-ZMBH Alliance, Im Neuenheimer Feld 282, 69120 Heidelberg, Germany

[5] German Cancer Research Center (DKFZ), Im Neuenheimer Feld 280, 69120 Heidelberg, Germany

[6] Institute for Cyber Science, Pennsylvania State University, University Park, PA, 16802, USA

* To whom correspondence should be addressed. Tel: (814) 867-5100; Fax: (814) 865-2927; Email: epo2@psu.edu

‡ These authors contributed equally to this work.

[#] Present address: Department of Physics, Indian Institute of Technology, Jammu 181121, India

[&] Present address: Department of Neurology, Columbia University College of Physicians and Surgeons, New York, NY, USA

S1 Text

Supplementary Methods

Derivation for the analytical expression for the translation-initiation rate. We derived an expression for translation-initiation rate under steady-state conditions (Eq. (1)). To do this, we equated Eqs. (2) and (3), which is valid according to Eq. (1), and then solved for $\omega(j,i)$, yielding

$$\omega(j,i) = \frac{\alpha(i)\left[1-\sum_{k=2}^{\ell+1}\rho(k,i)\right]}{\rho(j,i)f(j,j+\ell,i)}. \quad \text{(S1)}$$

Excluded volume interactions between ribosomes translating the same mRNA molecule increases the average amount of time they spend at each codon position. Therefore, the effective rate at which the jth codon position is translated by a ribosome decreases from $\omega(j,i)$ to $\omega(j,i)f(j,j+\ell,i)$ because a ribosome sitting at the $(j+\ell)^{th}$ codon position blocks the forward movement of the ribosome at the jth codon position. Thus, the average time, $\langle T(i)\rangle$, a ribosome takes to translate transcript i, composed of $N_c(i)$ codons, is

$$\langle T(i)\rangle = \sum_{j=2}^{N_C(i)} \frac{1}{\omega(j,i)f(j,j+\ell,i)}, \quad \text{(S2)}$$

where $f(j,j+\ell,i)$ =1 when $j > N_C(i) - 10$. Substituting $\omega(j,i)$ from Eq. (S1) into Eq. (S2),

$$\langle T(i)\rangle = \sum_{j=2}^{N_C(i)} \frac{1}{\frac{\alpha(i)\left[1-\sum_{k=2}^{\ell+1}\rho(k,i)\right]}{\rho(j,i)f(j,j+\ell,i)}f(j,j+\ell,i)} \quad \text{(S3)}$$

In Eq. (S3), we see that the term $f(j,j+\ell,i)$ cancels out in the denominator. Solving Eq. (S3) for $\alpha(i)$ yields

$$\alpha(i) = \frac{<\rho(i)>(N_c(i)-1)}{<T(i)>[\,1-\sum_{k=2}^{\ell+1}\rho(k,i)]}, \quad \text{(4)}$$

where $\langle\rho(i)\rangle = \frac{\sum_{j=2}^{N_c(i)}\rho(j,i)}{N_c(i)-1}$ is the average ribosome density of the ith transcript.

Estimation of $\langle\rho(i)\rangle$, $\rho(j,i)s$ and $\langle\tau(i)\rangle$. Measuring translation-initiation rates using Eq. (4) requires knowledge of $\langle\rho(i)\rangle$, $\rho(j,i)s$ and $\langle\tau(i)\rangle$, which we calculated using a combination of ribosome profiling, RNA-Seq and polysome profiling data. To calculate $\langle\rho(i)\rangle$ we used the experimental observation that the number of RNA-Seq reads aligned to a transcript is proportional to that transcript's copy number ($n_m(i)$) and coding sequence length [1]. We also make the conventional assumption that the number of ribosome profiling reads aligned to a transcript is proportional to the total number of ribosomes ($n_R(i)$) translating that transcript [2]. Therefore,

$$d(i) = a_1 n_m(i) N_c(i) \tag{S4}$$

and

$$c(i) = a_2 n_R(i), \tag{S5}$$

where $d(i)$ and $c(i)$ are the reads aligned to transcript i in RNA-Seq and ribosome profiling experiments, respectively, and a_1 and a_2 are proportionality constants. Dividing Eq. (S5) by (S4) yields the average ribosome density per codon on transcript i

$$\langle\rho(i)\rangle = \frac{n_R(i)}{n_m(i)N_c(i)} = \frac{a_1}{a_2}\frac{c(i)}{d(i)}. \tag{S6}$$

The ratio $\frac{c(i)}{d(i)}$ in Eq. (S6) is proportional to translation efficiency ($TE(i)$), defined as the ratio of ribosome profiling to RNA-Seq experiment reads in units of per kilobase, per million reads mapped to transcript i [3]. Introducing terms to get $\langle\rho(i)\rangle$ in terms of the TE we get,

$$\langle\rho(i)\rangle = \frac{a_1}{a_2}\frac{c(i)}{d(i)}\frac{10^9 N_C(i)}{10^9 N_C(i)}\frac{\frac{R^{Total}_{Ribo-Seq}}{R^{Total}_{Ribo-Seq}}}{\frac{R^{Total}_{NA-Seq}}{R^{Total}_{RNA-Seq}}} = \frac{a_1}{a_2}\frac{R^{Total}_{Ribo-Seq}}{R^{Total}_{NA-Seq}}\frac{\frac{c(i)10^9}{N_C(i)R^{Total}_{Ribo-Seq}}}{\frac{d(i)10^9}{N_C(i)R^{Total}_{RNA-Seq}}} = \frac{a_1}{a_2}\frac{R^{Total}_{Ribo-Seq}}{R^{Total}_{RNA-Seq}}\frac{RPKM_{Ribo-Seq}(i)}{RPKM_{RNA-Seq}(i)} \tag{S7}$$

Thus,

$$\langle\rho(i)\rangle = \xi\, TE(i), \tag{S8}$$

where $\xi = \frac{a_1}{a_2} \times \frac{R^{Total}_{Ribo-Seq}}{R^{Total}_{RNA-Seq}}$. $R^{Total}_{Ribo-Seq}$ and $R^{Total}_{RNA-Seq}$ are total number of reads mapped in the respective experiments which were used for calculation of RPKM values for transcript i and subsequently translational efficiency (TE).

As pointed out in Ref. [4], the ξ can be determined from the best fit line to the $\langle\rho(i)\rangle$ from polysome profiling (Ref. [5]) versus the $TE(i)$ calculated from ribosome profiling and RNA-Seq data (Ref. [3]). We carried out this analysis and find a statistically significant correlation between $\langle\rho(i)\rangle$ and $TE(i)$ (S12A Fig, Pearson $r = 0.51$, p value $\leq 10^{-60}$) with $\xi = 0.015$. Similar values of ξ are found using all combinations of ribosome profiling and RNA-Seq data [6] as well as polysome profiling data [7] (S12 Fig). With this value of ξ, we can use Eq. (S8) to calculate $\langle\rho(i)\rangle$ for any transcript, even those not in the original polysome profiling data set.

The $\rho(j,i)$s can be calculated by multiplying the probability that given a ribosome is translating transcript i it will be found at codon positon j (*i.e.*, $\frac{c(j,i)}{c(i)}$) by the average number of transcript i (*i.e.*, $\langle\rho(i)\rangle(N_c(i)-1)$)

$$\rho(j,i) = \frac{c(j,i)}{c(i)}\langle\rho(i)\rangle(N_c(i)-1). \tag{S9}$$

We estimated the synthesis time of a protein by using the finding that it scales linearly with the number of elongating codons in a transcript [8]

$$\langle T(i)\rangle = (N_c(i)-1)\langle\tau^{\mathrm{A}}\rangle. \tag{S10}$$

In Eq. (S9), $\langle\tau^{\mathrm{A}}\rangle$ is the transcriptome-wide average codon translation time. This approximation is supported both by experimental results [9] and a theoretical analysis that indicates this estimate is typically within 5% of the true synthesis time [8].

Thus, all the terms on the right hand side of Eq. (4) can be determined by utilizing data from ribosome profiling, RNA-Seq and polysome profiling, allowing for the determination of the initiation rate of each transcript.

Calculation of $\nabla \cdot \boldsymbol{J}$ in Eq. (5). We use the fundamental theorem of divergence to derive an expression for $\nabla \cdot \boldsymbol{J}$ describing the flow of ribosomes during translation elongation. According to the fundamental theorem of divergence, the volume integral of the divergence of a quantity over a region is equal to the surface integral of the same quantity [10]. That is,

$$\int (\nabla \cdot \boldsymbol{J}) dV = \int \boldsymbol{J} \cdot d\boldsymbol{A}. \tag{S11}$$

Assume that a given mRNA transcript, whose $\nabla \cdot \boldsymbol{J}$ we wish to calculate, is surrounded by a cube. The length, width and height of this hypothetical cube is L (codons). The mRNA transcript in this cube can be thought as a one-dimensional object lying along the x-axis. This means that under steady-state conditions, the y and z components of J are zero. Eq. (S11) for this hypothetical cube reduces to

$$\int_0^L \int_0^L \int_0^L \nabla \cdot \boldsymbol{J}\, dx\, dy\, dz = \int_0^L \int_0^L J dy dz. \tag{S12}$$

Further simplification of Eq. (S12) yields

$$\int_0^L \nabla \cdot \boldsymbol{J}\, dx = J. \tag{S13}$$

Under steady state conditions, ribosome flux at each codon position is the same. This means J is independent of the x-axis. Thus,

$$\nabla \cdot \boldsymbol{J} = \frac{J}{L} \tag{S14}$$

Estimation of $\frac{\rho(t=\Delta t, L)}{\rho(t=0, L)}$ for use in Eq. (6). Calculation of the transcriptome-wide average elongation rate requires the use of relative ribosome density $\frac{\rho(t=\Delta t, L)}{\rho(t=0, L)}$ in Eq. (6), which we calculated from the meta-gene analysis of the ribosome run-off experimental data. To do that first we calculated the average ribosome density at each codon position as

$$\overline{R_T}(j, \Delta t) = \frac{\sum_i R(i,j,\Delta t)}{N_t(j)}\,, \tag{S15}$$

where $R(i, j, \Delta t)$ is the ribosome profiling reads aligned at codon position j on transcript i divided by the average ribosome profiling reads per codon in that transcript in a sample with the run-off time Δt, and $N_t(j)$ is the number of mRNA transcripts identified in the experiment whose CDS length is either equal or greater than j codons. The total number of aligned ribosome profiling reads in a sample can vary from one experiment to the next, therefore we further normalized $\overline{R_T}(j, \Delta t)$ by dividing this to its average value calculated in the region of the meta-gene profile in which no depletion of ribosome reads occurs at the longest run-off time. For example, we normalized $\overline{R_T}(j, \Delta t)$ in mouse embryonic stem cells runoff data by dividing to the average value of $\overline{R_T}(j, \Delta t)$ between codon positions 800 and 1,000 (Fig. 2).

We assume that the number of ribosome profiling reads aligned to a transcript is proportional to the number of ribosomes translating that transcript. Under this assumption, the number of normalized average ribosome profiling reads $\overline{R_T}(j, \Delta t)$ within the first L codon positions of the transcripts is proportional to $\rho(t = \Delta t, L)$. Therefore,

$$\frac{\rho(t=\Delta t, L)}{\rho(t=0, L)} = \frac{\sum_{j=2}^{L} \overline{R_T}(j,\Delta t)}{\sum_{j=2}^{L} \overline{R_T}(j,0)}. \tag{S16}$$

This expression for $\frac{\rho(t=\Delta t, L)}{\rho(t=0, L)}$ is then used in Eq. (6) to calculate the transcriptome-wide average elongation rate.

Derivation of Eq. (10) from Eq. (8) and Eq. (9): Eq. (8), restated below, defines the steady state condition of translation. Eq. (9) is the mean synthesis time of transcript i, which is the sum of the translation times of the elongating codons of transcript i.

$$\frac{N_{2,i}^{\text{ribo}}}{\tau(2,i)} = \frac{N_{3,i}^{\text{ribo}}}{\tau(3,i)} = \cdots \frac{N_{j,i}^{\text{ribo}}}{\tau(j,i)} = \cdots = \frac{N_{N_c(i),i}^{\text{ribo}}}{\tau(N_c(i),i)} \tag{8}$$

$$\langle T(i) \rangle = \tau(2,i) + \tau(3,i) + \cdots + \tau(N_c(i),i) \tag{9}$$

The translation time of a codon position l in transcript i can be expressed (through a simple algebraic rearrangement of Eq. (8)) in terms of the translation time of any other codon position j as

$$\tau(l,i) = \frac{\tau(j,i) N_{il}^{\text{Ribo}}}{N_{ij}^{\text{Ribo}}}. \tag{S17}$$

For each codon position, $l = 2, 3, 4, \ldots., N_c(i)$, we substitute Eq. (S17) into Eq. (9), which yields

$$\langle T(i) \rangle = \frac{\tau(j,i) N_{i,2}^{\text{Ribo}}}{N_{ij}^{\text{Ribo}}} + \frac{\tau(j,i) N_{i,3}^{\text{Ribo}}}{N_{ij}^{\text{Ribo}}} + \cdots + \frac{\tau(j,i) N_{iN_i^c}^{\text{Ribo}}}{N_{ij}^{\text{Ribo}}}. \tag{S18}$$

We then pull out the common terms, yielding

$$\langle T(i) \rangle = \frac{\tau(j,i)}{N_{ij}^{\text{Ribo}}} \left[N_{i,2}^{\text{Ribo}} + N_{i,3}^{\text{Ribo}} + \cdots + N_{iN_i^c}^{\text{Ribo}} \right], \tag{S19}$$

where the term in square brackets on the right-hand-side of Eq. (S19) can be expressed as a summation, yielding

$$\langle T(i) \rangle = \frac{\tau(j,i)}{N_{ij}^{\text{Ribo}}} \sum_{l=2}^{N_c(i)} N_{l,i}^{\text{ribo}}. \tag{S20}$$

Rearranging Eq. (S20) yields Eq. (10) in the main text:

$$\tau(j,i) = \frac{N_{j,i}^{\text{ribo}}}{\sum_{l=2}^{N_c(i)} N_{l,i}^{\text{ribo}}} \langle T(i) \rangle\ . \tag{10}$$

Supplementary References

1. Nagalakshmi U, Waern K, Snyder M. RNA-seq: A method for comprehensive transcriptome analysis. Current Protocols in Molecular Biology. 2010. p. 4.11.1-4.11.13. doi:10.1002/0471142727.mb0411s89

2. Ingolia NT, Brar GA, Rouskin S, McGeachy AM, Weissman JS. The ribosome profiling strategy for monitoring translation in vivo by deep sequencing of ribosome-protected mRNA fragments. Nat Protoc. 2012;7: 1534–1550. doi:10.1038/nprot.2012.086

3. Weinberg DE, Shah P, Eichhorn SW, Hussmann JA, Plotkin JB, Bartel DP. Improved Ribosome-Footprint and mRNA Measurements Provide Insights into Dynamics and Regulation of Yeast Translation. Cell Rep. The Authors; 2016;14: 1787–1799. doi:10.1016/j.celrep.2016.01.043

4. Dao Duc K, Song YS. The impact of ribosomal interference, codon usage, and exit tunnel interactions on translation elongation rate variation. PLoS Genet. 2018;14: e1001508. doi:10.1371/journal.pgen.1007166

5. MacKay VL, Li X, Flory MR, Turcott E, Law GL, Serikawa KA, et al. Gene Expression Analyzed by High-resolution State Array Analysis and Quantitative Proteomics. Mol Cell Proteomics. 2004;3: 478–489. doi:10.1074/mcp.M300129-MCP200

6. Nissley DA, Sharma AK, Ahmed N, Friedrich UA, Kramer G, Bukau B, et al. Accurate prediction of cellular co-translational folding indicates proteins can switch from post- to co-translational folding. Nat Commun. 2016;7: 10341. doi:10.1038/ncomms10341

7. Arava Y, Wang Y, Storey JD, Liu CL, Brown PO, Herschlag D. Genome-wide analysis of mRNA translation profiles in Saccharomyces cerevisiae. Proc Natl Acad Sci U S A. 2003;100: 3889–3894. doi:10.1073/pnas.0635171100

8. Sharma AK, Ahmed N, O'Brien EP. Determinants of translation speed are randomly distributed across transcripts resulting in a universal scaling of protein synthesis times. Phys Rev E. American Physical Society; 2018;97: 22409. Available: https://link.aps.org/doi/10.1103/PhysRevE.97.022409

9. Ingolia NT, Lareau LF, Weissman JS. Ribosome profiling of mouse embryonic stem cells reveals the complexity and dynamics of mammalian proteomes. Cell. Elsevier Inc.; 2011;147: 789–802. doi:10.1016/j.cell.2011.10.002

10. Griffiths DJ. Introduction to electrodynamics, 3rd Ed. Prentice Hall. 1999. doi:10.1016/S1570-8659(04)13001-9
