# Supplementary figures and images for: A chemical kinetic basis for measuring translation initiation and elongation rates from ribosome profiling data
Source: PLoS Comput Biol. 2019 May 23;15(5):e1007070. doi: 10.1371/journal.pcbi.1007070 (PMC6559674; doi:10.1371/journal.pcbi.1007070)

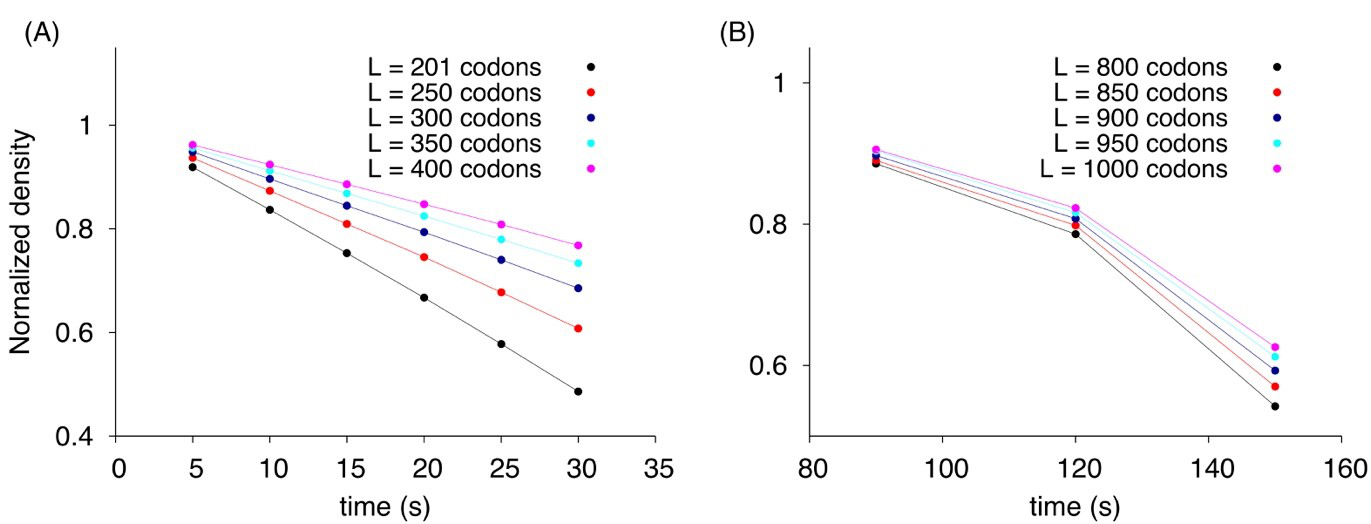

Supplement: S1 Fig — (A) The normalized ribosome read density in S. cerevisiae using in silico run-off experiment data in the first 201, 250, 300, 350 and 400 codons are plotted as a function of time. (B) The normalized ribosome read density in mouse stem cells (Ref. [26]) are plotted as a function of time in the first 800, 850, 900, 950 and 1000 codons. Lines are to guide the eye. (TIFF) [file pcbi.1007070.s002.tiff]

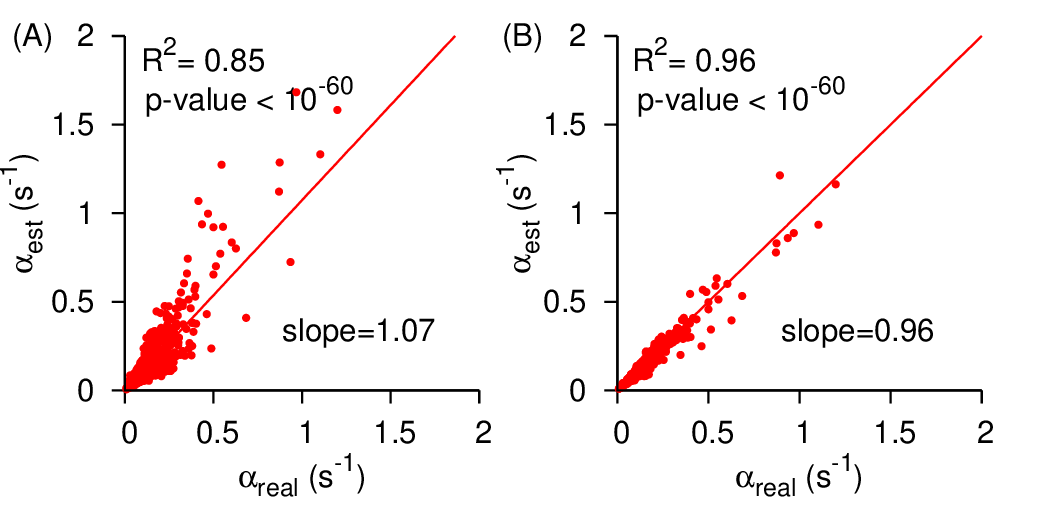

Supplement: S2 Fig — (A) Translation-initiation rates determined by applying Eq (4) to simulated ribosome profiling data are plotted against the actual initiation rates used in the simulations. These initiation rates were calculated using Eq. (S10) for the protein synthesis times. (B) Same as (A) but the average protein synthesis times were measured from our simulations of the translation process. The solid lines in (A) and (B) are the lines of the best fit. (TIFF) [file pcbi.1007070.s003.tiff]

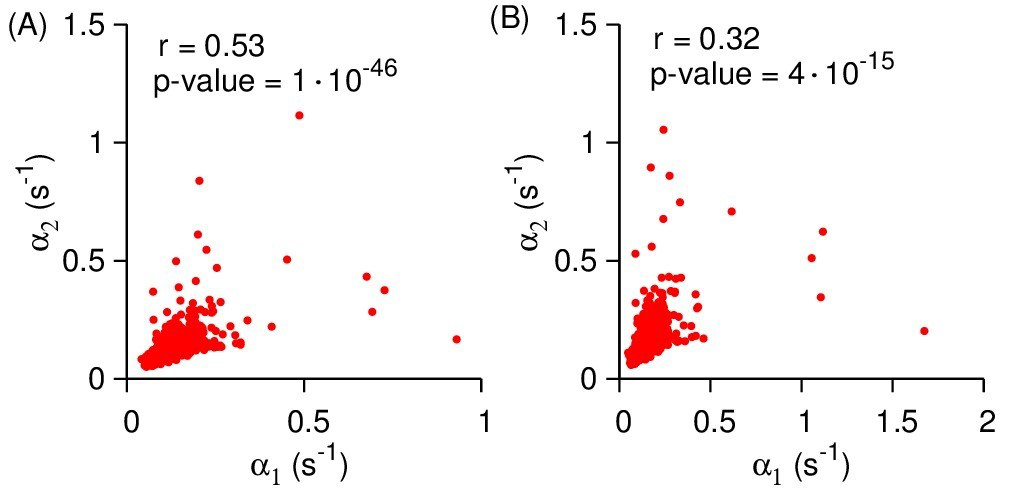

Supplement: S3 Fig — α1 and α2 are the translation-initiation rate calculated by Eq (4) using the ribosome profiling and RNA-Seq data reported in Nissley et al. [43] and Weinberg et al. [16], respectively. Polysome profiling data reported in Mackay et al. [42] and Arava et al. [29] were used in (A) and (B), respectively. (TIFF) [file pcbi.1007070.s004.tiff]

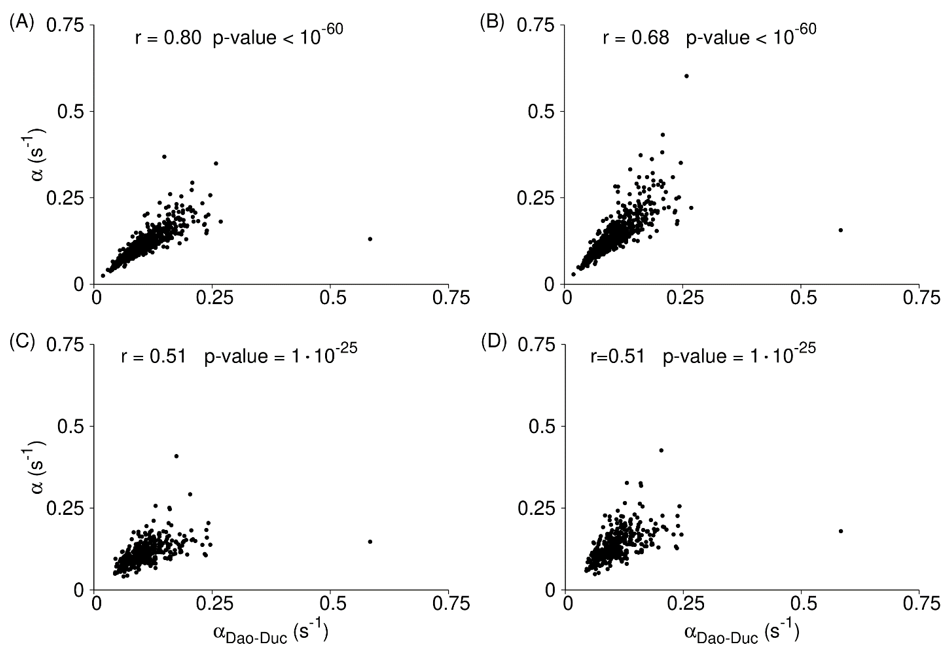

Supplement: S4 Fig — In vivo initiation rates calculated by Eq (4) using the ribosome profiling and RNA-seq data from Weinberg et al. [16] were compared with the ones reported in Dao Duc and Song [14] in (A) and (C); In vivo initiation rates calculated by Eq (4) using the ribosome profiling and RNA-seq data from Nissley et al. [43] were compared with the ones reported in Dao Duc and Song [14] in (B) and (D). Polysome profiling data reported in Mackay et al. [42] were used to calculate in vivo initiation rates in (A) and (B); Polysome profiling data reported in Arava et al. [29] were used to calculate in vivo nitiation rates in (C) and (D). (TIFF) [file pcbi.1007070.s005.tiff]

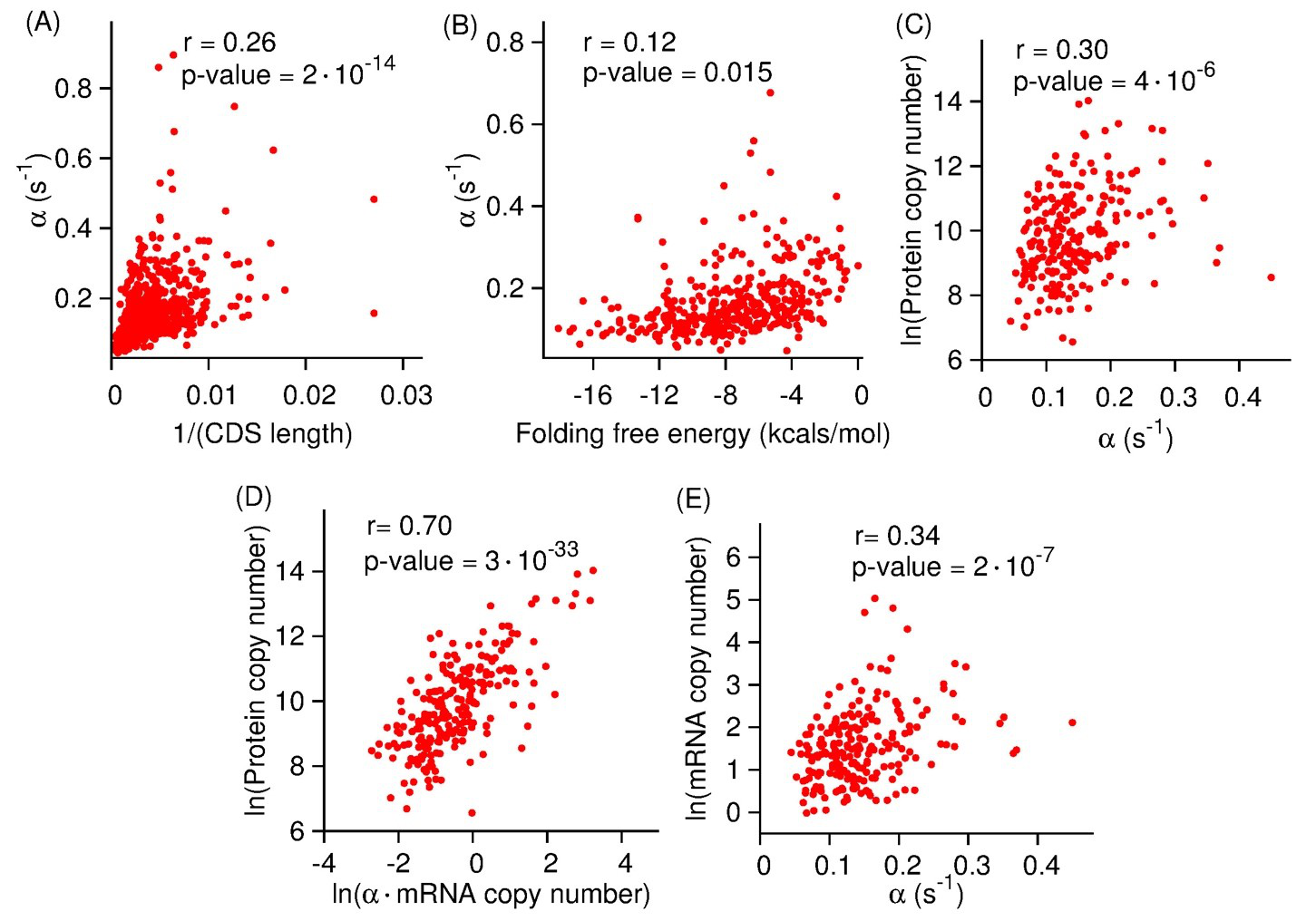

Supplement: S5 Fig — In vivo translation-initiation rates of S. cerevisiae transcripts are plotted against the inverse of their CDS length, folding energy of mRNA molecule near the 5′ cap and protein copy number in (A), (B) and (C), respectively. (D) The copy number of S. cerevisiae proteins are plotted as a function of the product of the initiation rate of transcripts that encode them and that transcript’s copy number in a cell. (E) mRNA copy number is plotted against the translation-initiation rate. (TIFF) [file pcbi.1007070.s006.tiff]

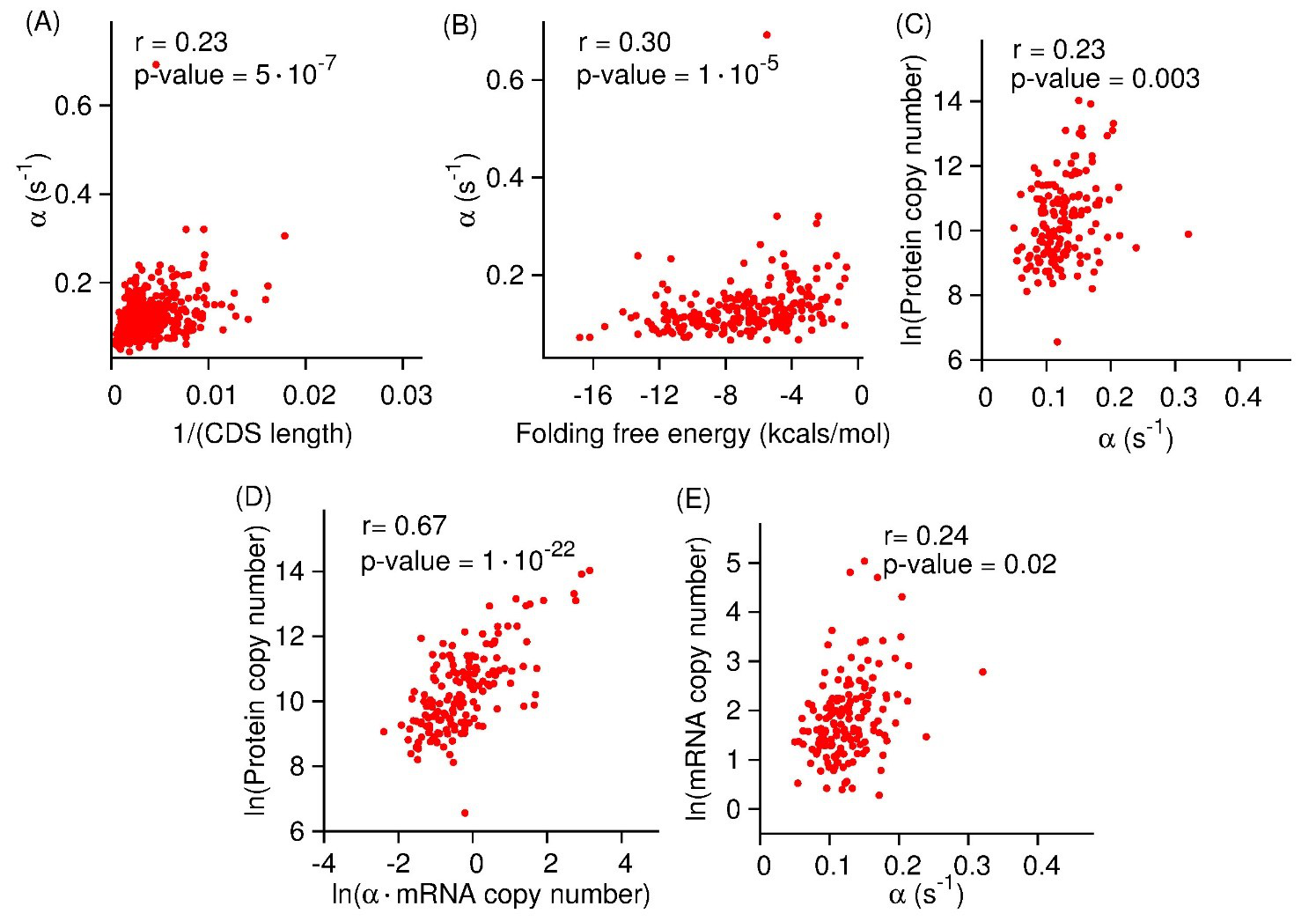

Supplement: S6 Fig — In vivo translation-initiation rates of S. cerevisiae transcripts are plotted against the inverse of their CDS length, folding energy of mRNA molecule near the 5′ cap and protein copy number in (A), (B) and (C), respectively. (D) The copy number of S. cerevisiae proteins are plotted as a function of the product of the initiation rate of transcripts that encode them and that transcript’s copy number in a cell. (E) mRNA copy number is plotted against the translation-initiation rate. (TIFF) [file pcbi.1007070.s007.tiff]

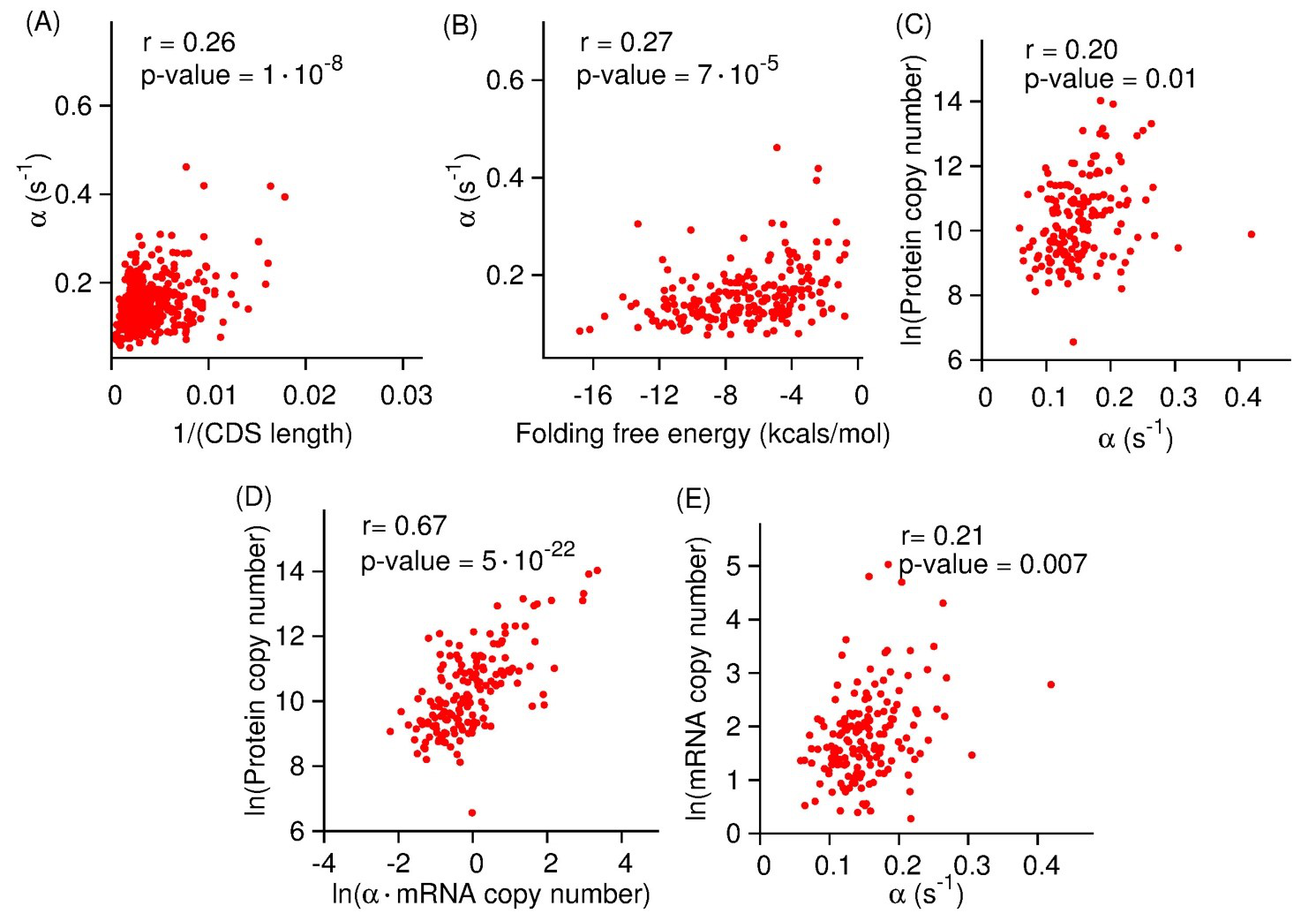

Supplement: S7 Fig — In vivo translation-initiation rates of S. cerevisiae transcripts are plotted against the inverse of their CDS length, folding energy of mRNA molecule near the 5′ cap and protein copy number in (A), (B) and (C), respectively. (D) The copy number of S. cerevisiae proteins are plotted as a function of the product of the initiation rate of transcripts that encode them and that transcript’s copy number in a cell. (E) mRNA copy number is plotted against the translation-initiation rate. (TIFF) [file pcbi.1007070.s008.tiff]

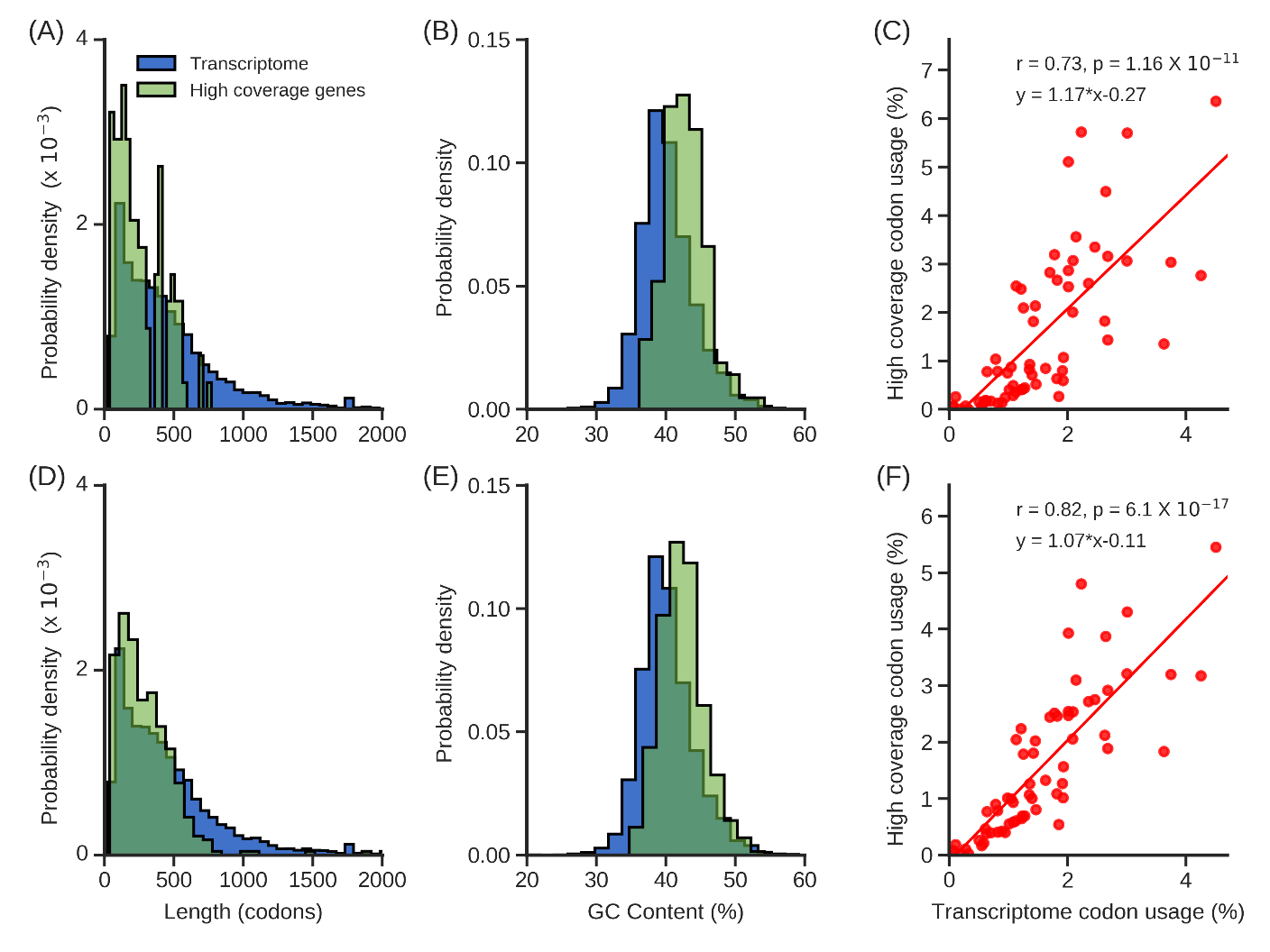

Supplement: S8 Fig — Probability distributions of CDS length and percent GC content from the data set of 117-transcripts from Ref. [43] (green) and from the entire transcriptome (blue) are plotted in (A) and (B), respectively. (C) Scatter plot of the codon usage in the whole genome versus the 117-transcript data set from Ref. [43]. (D), (E) and (F) are the same as (A), (B) and (C), respectively, except 364-transcripts from Ref. [53] is used. (TIFF) [file pcbi.1007070.s009.tiff]

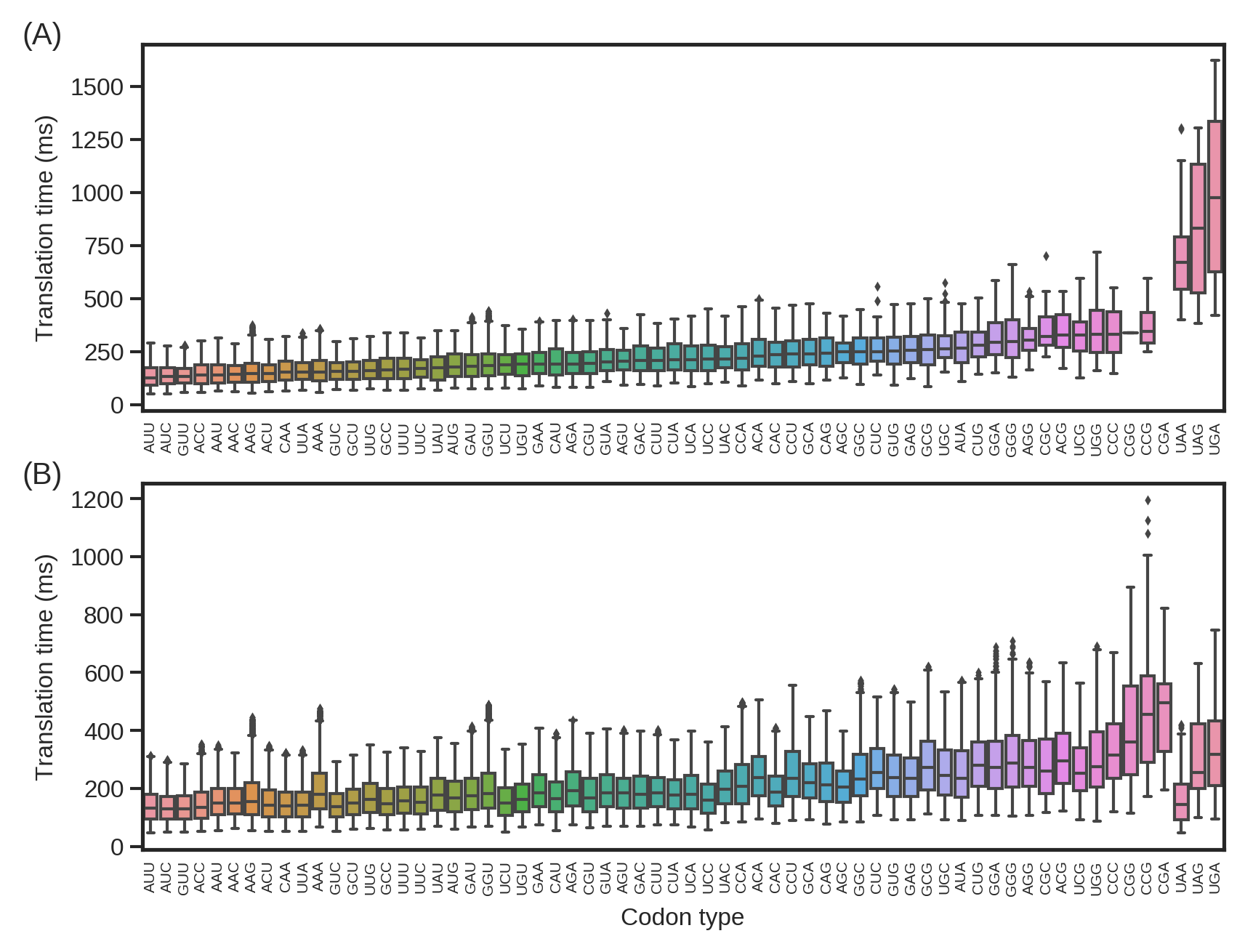

Supplement: S9 Fig — (A) The translation time distributions for each codon type is shown for the dataset of Nissley et al. [43]. The distribution is shown ignoring the extreme 5th percentiles at both ends of the distribution. The codons are sorted based on the medians of their translation time distributions. There are only three instances of CGG and one instance of CGA in our gene subset and hence their boxplot is not noticeable. (B) Same as (A) but for the dataset of Williams et al. [53]. The sorting is the same as in (A). (TIFF) [file pcbi.1007070.s010.tiff]

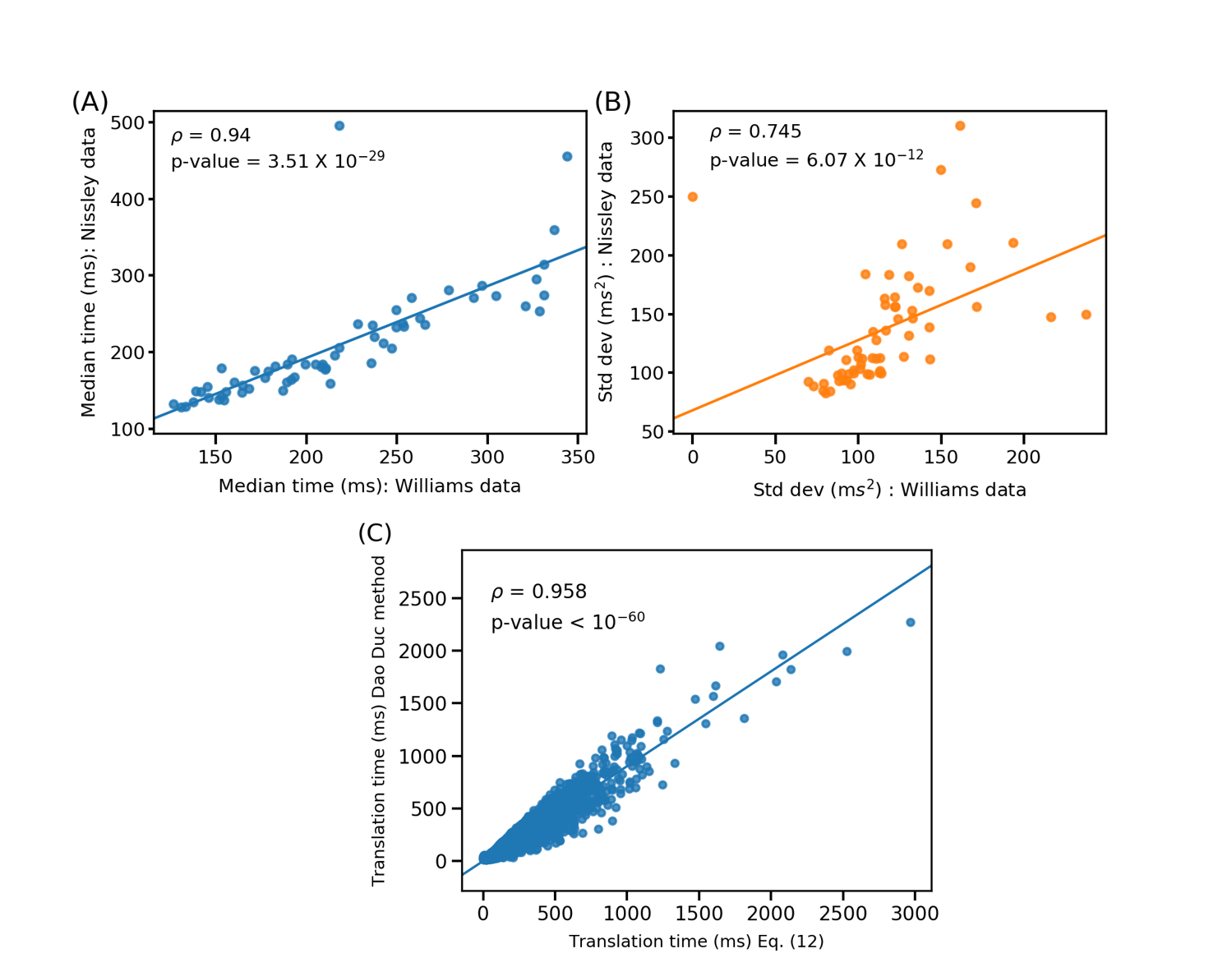

Supplement: S10 Fig — (A) The medians of the translation time distributions of the 64 codon types are highly correlated between the datasets of Nissley et al. [43] and Williams et al. [53]. (B) The standard deviations of these translation time distributions are also highly correlated for the two datasets indicating that the variability of translation times is reproducible across datasets. (C) The codon translation rates obtained using Eq (12) for the dataset from Weinberg et al. [16] is correlated with codon translation rates inferred in the study of Dao Duc and Song [14] on the same dataset. (TIFF) [file pcbi.1007070.s011.tiff]

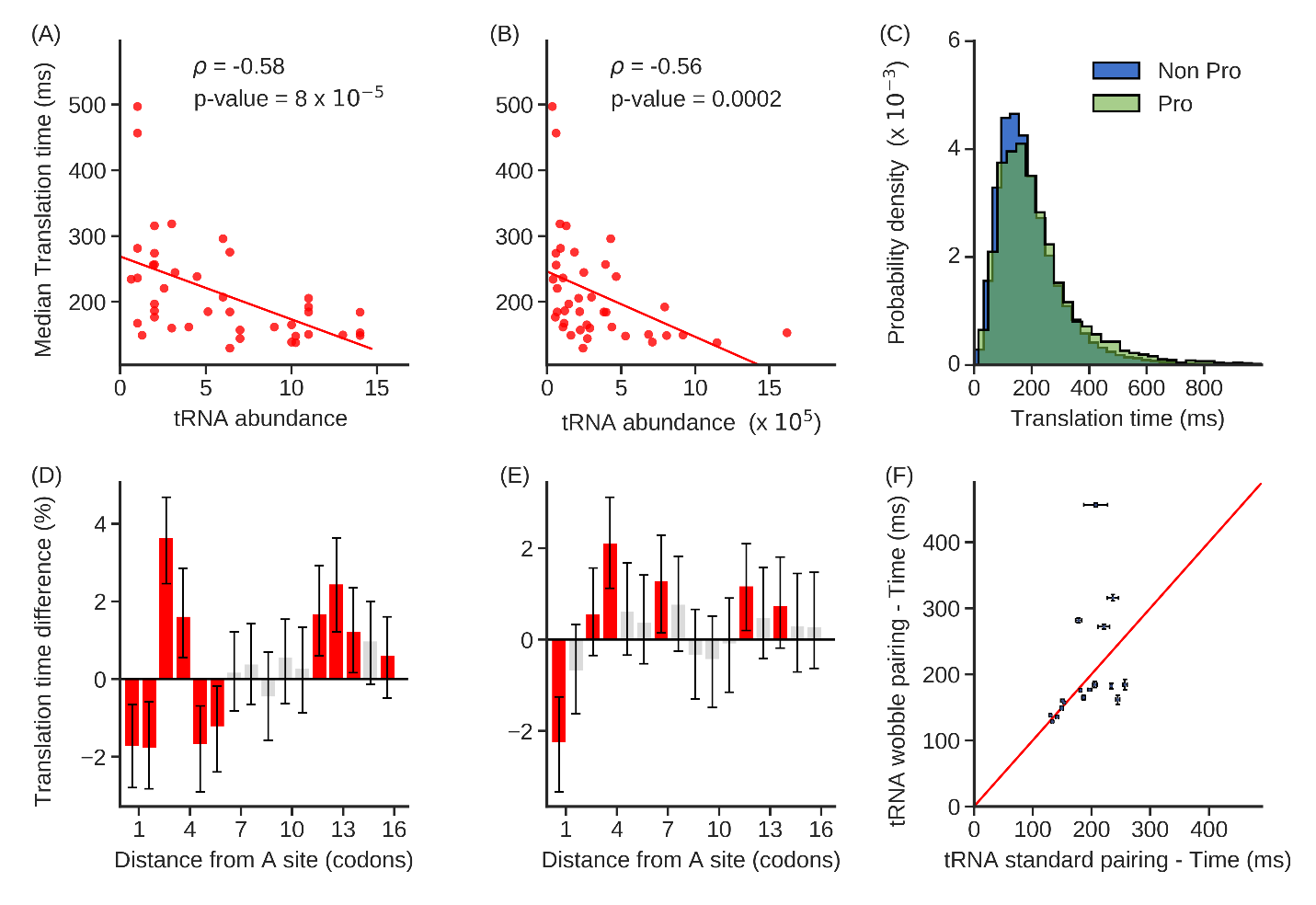

Supplement: S11 Fig — (A-B) Median translation times of codon types are negatively correlated with cognate tRNA abundance estimated by (A) gene copy number and (B) RNA-Seq gene expression. (C) Probability distribution of translation time of codons in the A-site either when a proline is present in the P-site (green) or when a proline is not present in the P-site (blue). (D-E) Percentage difference in median translation times when mRNA structure is present relative to when it is not present as a function of codon position after the A-site. Grey bars indicate results that are not statistically significant. Error bars are the 95% C.I. calculated using 104 bootstrap cycles; significance is assessed using the Mann-Whitney U test corrected with the Benjamini Hochberg FDR method for multiple-hypothesis correction. mRNA structure information used in (D) and (E) were taken from in vivo DMS and in vitro PARS data, respectively. (F) Scatter plot of the median translation times of pairs of codon types that are decoded by the same tRNA molecule. The red line is the identity line. The list of tRNA molecules and which codon they decode were taken from Ref. [54]. Error bars are standard error about the median calculated with 104 bootstrap cycles. (TIFF) [file pcbi.1007070.s012.tiff]

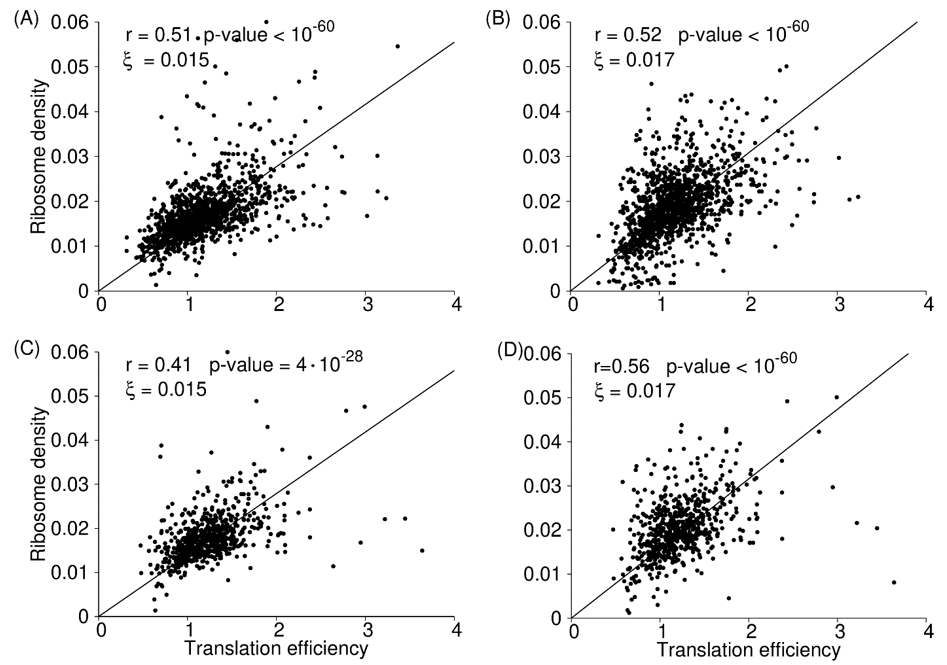

Supplement: S12 Fig — Translation efficiency in (A) and (B) are calculated using the ribosome profiling and RNA-Seq data reported in Ref. [16]; Translation efficiency in (C) and (D) are calculated using ribosome profiling and RNA-Seq data reported in Ref. [43]. Ribosome density used in (A) and (C) are from the polysome profiling data reported in Ref. [42] whereas the ribosome density in (B) and (D) are provided by Ref. [29]. The solid line in all these figures represent the best fit of y = ξx line. (TIFF) [file pcbi.1007070.s013.tiff]
