# Supplementary material for: A chemical kinetic basis for measuring translation initiation and elongation rates from ribosome profiling data
Source: PLoS Comput Biol. 2019 May 23;15(5):e1007070. doi: 10.1371/journal.pcbi.1007070 (PMC6559674; doi:10.1371/journal.pcbi.1007070)
Supplement: S1 Table — For all possible combinations of ribosome profiling and RNA-Seq data [16,43] and polysome profiling data [29,42], we see a consistent result that the median translation-initiation rate is lower for transcripts with at least one uAUG. The result is not statistically significant for combination of Refs. [43] and [42] (p-value = 0.052). (PDF) [file pcbi.1007070.s014.pdf]

S1 Table: Transcripts containing at least one upstream AUG (uAUG) have lower median translation initiation rates. For all possible combinations of ribosome profiling and RNA-Seq data [16,43] and polysome profiling data [29,42], we see a consistent result that the median translation initiation rate is lower for transcripts with at least one uAUG. The result is not statistically significant for combination of Refs. [43] and [42] (p-value =0.052)

**Dataset Refs**	**Total transcripts with initiation rates**	**Number of transcripts (uAUG)**	**Number of transcripts (non uAUG)**	**Transcripts with no 5' UTR annotation**	**Median initiation rate (uAUG) (s^{-1})**	**Median initiation rate (non uAUG) (s^{-1})**	**Mann Whitney U test p-value**
[16] and [42]	1287	45	997	245	0.095	0.112	**0.006**
[16] and [29]	1249	42	972	235	0.115	0.134	**0.009**
[43] and [42]	652	16	529	107	0.104	0.119	0.052
[43] and [29]	643	17	518	108	0.121	0.145	**0.025**
