# Supplementary material for: A chemical kinetic basis for measuring translation initiation and elongation rates from ribosome profiling data
Source: PLoS Comput Biol. 2019 May 23;15(5):e1007070. doi: 10.1371/journal.pcbi.1007070 (PMC6559674; doi:10.1371/journal.pcbi.1007070)
Supplement: S2 Table — For all possible combinations of ribosome profiling and RNA-Seq data [16,43] and polysome profiling data [29,42], we see a consistent result that the median translation-initiation rate is higher for transcripts with sequence context similar to Kozak sequence (see Methods for details). The result for combination of Refs [43] and [29] is however not statistically significant (p-value = 0.065). (PDF) [file pcbi.1007070.s015.pdf]

S2 Table: Transcripts with sequence context around start codon similar to Kozak sequence have higher translation initiation rates. For all possible combinations of ribosome profiling and RNA-Seq data [16,43] and polysome profiling data [29,42], we see a consistent result that the median translation initiation rate is higher for transcripts with sequence context similar to Kozak sequence (see Methods for details). The result for combination of Refs [43] and [29] is however not statistically significant (p-value = 0.065)

**Dataset Refs**	**Number of transcripts (similar to Kozak sequence)**	**Number of transcripts (dissimilar to Kozak sequence)**	**Median initiation rate (similar to Kozak sequence) (s^{-1})**	**Median initiation rate (dissimilar to Kozak sequence) (s^{-1})**	**Mann Whitney U test p-value**
[16] and [42]	201	202	0.116	0.100	**0.005**
[16] and [29]	197	193	0.138	0.123	**0.012**
[43] and [42]	127	74	0.121	0.112	**0.034**
[43] and [29]	126	70	0.146	0.136	0.065
